# Diets Containing Sea Cucumber (*Isostichopus badionotus*) Meals Are Hypocholesterolemic in Young Rats

**DOI:** 10.1371/journal.pone.0079446

**Published:** 2013-11-19

**Authors:** Leticia Olivera-Castillo, Alberto Davalos, George Grant, Nina Valadez-Gonzalez, Jorge Montero, Hirian Alonso Moshe Barrera-Perez, Yasser Chim-Chi, Miguel Angel Olvera-Novoa, Víctor Ceja-Moreno, Pablo Acereto-Escoffie, Jorge Rubio-Piña, Rossanna Rodriguez-Canul

**Affiliations:** 1 Centro de Investigacion y de Estudio Avanzados del Instituto Politecnico Nacional - Unidad Merida, Merida, Yucatan, Mexico; 2 Instituto Madrileño de Estudios Avanzados - Alimentación, Campus de Excelencia Internacional Universidad Autonoma de Madrid+Consejo Superior de Investigaciones Cientificas, Madrid, Spain; 3 Rowett Institute of Nutrition and Health, University of Aberdeen, Aberdeen, Scotland; 4 Centro de Investigaciones Regionales Dr. Hideyo Noguchi, Universidad Autonoma de Yucatan, Merida, Yucatan, Mexico; 5 Laboratorio de Anatomia Patologica, Merida, Yucatan, Mexico; 6 Facultad de Ingenieria Quimica, Universidad Autonoma de Yucatan, Merida, Yucatan, Mexico; Clermont Université, France

## Abstract

Sea cucumber is widely consumed as a putative functional food. It contains many biologically-active substances, but only limited research on its properties *in vivo* has been done. The effects of different meals containing *Isostichopus badionotus*, a sea cucumber from southeast Mexico, on growth performance and body lipid profile in young rats were analyzed. Sea cucumber body wall was either lyophilized, cooked (100 °C, 1 h in water) and lyophilized, or oven-dried (70 °C for 12 h). It was then ground and incorporated into cholesterol-containing diets. I. badionotus meals supported growth and improved lipid profile in rats. In particular, serum cholesterol, low density lipoproteins, triglycerides concentration and atherogenic index values were greatly reduced by some *I. badionotus* containing diets. Liver total lipids, triglycerides and cholesterol were also reduced. Cooking or heat-treatment of the meals lowered but did not abolish their hypolipidemic potency. Gene expression analysis of several key genes involved in cholesterol and lipid metabolism in liver showed that diets containing *I. badionotus* repressed the induction of key genes associated with dyslipidemia exerted by cholesterol supplementation. Consumption of *I. badionotus* from the Yucatan Peninsula is beneficial for dyslipidemia, although biological effect is clearly dependent on preparation method.

## Introduction

Cardiovascular diseases (CVD) are a leading cause of mortality worldwide [1,2]. Their incidence is already high in most developed countries but is also increasing very rapidly in many developing regions [3]. Obesity and dyslipidemia are important risk factors for CVD [4]. It is estimated that 15% (33.6 million) of the adult population in the USA have greatly elevated (≥240 mg/dL) blood cholesterol [2]. The high prevalence of CVD is associated with major nutritional and lifestyle changes in the population, particularly the wider availability and consumption of fat- or energy-rich foods in conjunction with a more sedentary lifestyle. The risks of CVD can be reduced by switching from a diet high in mainly fat- and energy-rich foods to a more balanced one containing a mix of foodstuffs, including vegetables, fruit, fish, etc. [5,6]. In addition, the use of dietary supplements or functional foods that limit digestion / absorption of fat and carbohydrates or ameliorate adverse systemic effects of diet may aid in lowering CVD risk [2]. Indeed, the use of dietary supplements is now widespread, despite limited scientific evidence of their efficacy [2]. Identification of functional foods or dietary supplements that can be shown, under rigorous testing, to help in preventing, delaying or treating CVD would be a valuable addition to the treatment arsenal. 

**Table 1:** Composition of control and experimental diets fed rats.

	Diets^1^				
Constituents	CNC	CC	LWS50	CS50	OS50
	*g/ kg*				
Lactalbumin^2^	142.35	142.35	71.43	71.12	71.12
Sea cucumber meal^3^	---	---	86.63	91.60	83.33
Corn starch	534.20	514.20	486.25	480.37	488.98
Potato starch	100	100	100	100	100
Glucose	75	75	75	75	75
Corn oil	48.45	48.45	52.69	52.69	52.69
Minerals^4^	50	50	50	50	50
Vitamins^5^	50	50	50	50	50
Cholesterol^6^	----	20	20	20	20
L-tryptophan	---	---	2.5	2.5	2.5
L-lysine	---	---	4.20	2.82	4.4
L-methionine	---	---	5.40	5.5	5.4
Protein^7^	12	12	12	12	12
Available energy *MJ*	16.53	16.44	16.32	16.44	16.32

1 CNC = control with no added cholesterol; CC = Control with 2% cholesterol; LWS50 = 50% lyophilized and washed sea cucumber + CC; CS50 = 50% water-cooked sea cucumber + CC; OS50 = 50% oven-cooked sea cucumber + CC ^2^.Lactalbumin (composition: Protein 84 g/100 g, lipids 4.6 g/100 g ) ^3^.Sea cucumber meal (composition: protein 49-72.5 g/100 g; lipids 0.6 g/100 g) ^4^.Mineral mix (1kg): 400 mg copper sulfate; 5000 mg iron sulfate; 4000 mg manganese sulfate; 3600 mg zinc sulfate; 40 mg potassium iodine; 120 mg sodium fluoride; 10 mg ammonium vanadate; 80 mg nickel chloride; 120 mg stannous chloride; 6 mg sodium selenate; 960 mg chromium aluminum; 420 mg calcium carbonate; 314 g potassium dehydrogenate orthophosphate; 22 g potassium chloride; 102 g magnesium sulfate; 142 g disodium hydrogenated orthophosphate ^5^.Vitamin mix (1 kg): 200 mg thiamine; 200 mg pyridoxine; 200 mg riboflavin; 200 mg *p-*aminobenzoic acid; 600 mg nicotinic acid; 400 mg calcium pantothenate; 100 mg folic acid; 100 mg biotin; 8000 mg inositol; 5000 mg α-tocopherol; 230 mg retinylacetate; 300 mg cholecalcipherol; 5 mg cyanocobalamine; 100 mg menadione; and 20 g choline chloride. Weight completed to 1 kg with corn starch ^6^.Cholesterol (Sigma Mexico) ^7^.Protein = N x 6.25.

Sea cucumber is a benthic marine organism distributed worldwide, with the highest diversity in shallow tropical waters. It is widely consumed in East Asia, where it is considered to have significant health benefits [7]. An extensive worldwide commercial fishery exists to supply this market [8]. Three sea cucumber species can be found off the coasts of the Yucatan Peninsula in southeast Mexico [9]. Of special interest in this region is *Isostichopus badionotus*. It has a turgid body wall, a desirable trait on international markets, and is consequently harvested intensively.

Sea cucumber body wall consists mainly of collagen and mucopolysaccharides, but also contains potentially bioactive substances, such as triterpenes, sphingolipids [10]; antitumor agents, antioxidants [11]; opsonins [12]; lectins [13]; and glycosaminoglycans [14]. Although widely consumed as a functional food, it is not clear how or if consumption of sea cucumber or its bioactive components modulates body metabolism and health. In the present study, the effects of dietary intake of sea cucumber on lipid profile, metabolism and weight gain in young rats was evaluated.

**Table 2:** Proximate composition of sea cucumber (*I. badionotus*) meals.

	**Meals** ^1^			
	**LSM**	**LWSM**	**CSM**	**OSM**
**Parameters** ^2^	*g/ kg*			
Moisture	53.2 ± 15^a^	66 ± 15^a^	76 ± 5.0^b^	77 ± 10^b^
Ash	603.1 ± 61^a^	425 ± 61^b^	174.5 ± 98^d^	306.8 ± 28^c^
Crude lipids	6.29 ± 2.72^b^	6.3 ± 2.72^b^	6.5 ± 0.29^c^	6.7 ± 0.22^a^
Crude protein	367.0 ± 75.0^c^	490 ± 75.0^b^	725 ± 19.1^a^	587.5 ± 34.0^b^
NFE^3^	12.9 ± 7.50^b^	12.7 ± 7.50^b^	17 ± 2.71^b^	21.7 ± 7.00^a^
Amino Acids^4^		*g/100 g protein*		
Lysine		2.36	1.79	1.37
AAA		7.25	6.45	5.74
SAA		2.4	2.4	2.3
Threonine		5.5	5.28	4.23
Leucine		4.18	3.83	3.14
Isoleucine		2.6	3.02	2.52
Valine		3.88	3.77	3.13
Tryptophan		0.49	0.4	0.4
Arginine		8.0	8.6	6.41
Histidine		0.9	0.79	0.64
Aspartic acid		10	10.32	8.27
Glutamic acid		15.82	12.4	12.4
Serine		2.35	2.4	2.4
Glycine		21.5	22.7	21.5
Alanine		7.37	10.56	8.28
Proline		1.9	1.8	1.7
Chemical index		0.66	0.66	0.44
Lysine/Arginine		0.295	0.208	0.21

1 LSM = lyophilized sea cucumber meal; LWSM = lyophilized washed sea cucumber meal; CSM = water-cooked sea cucumber meal; OSM = oven-cooked sea cucumber meal^2^.Values are the mean of three replicates ± standard deviation; ^abcd^Different letter superscripts in the same row indicate significant difference (P<0.05) ^3^.NFE = Nitrogen Free Extract ^4^.AAA = total aromatic amino acids; SAA = total sulphur amino acids.

## Methods

### Organism Collection and Handling

Sea cucumber *Isostichopus badionotus* (Holothuria) were collected from the sea floor off the coast of Sisal, Yucatan state, Mexico. All required permits were valid at the time of collection (SAGARPA permit No. DGOPA/1009/210809/08761). Immediately upon removal from the sea floor, while still in marine water, the organisms were individually placed in plastic bags. When brought to the surface they were placed in marine water in coolers and kept at 22 to 24 °C, a temperature range similar to that of the collection site. Temperature was controlled to prevent proteolysis or autolysis. The organisms were then transported to the laboratory in Merida, Yucatan, and placed in tanks with marine water under controlled conditions (23-24 °C; 20 organisms/m^2^ stocking density). As soon as possible, the animals were removed, quickly eviscerated, leaving only the body wall, and washed with cold distilled water.

The sea cucumber body walls were prepared with three methods: lyophilized [LSM, lyophilized sea cucumber meal]; cooked in water for 1 h at 100 °C followed by lyophilization [CSM, cooked sea cucumber meal]; or oven-dried at 70 °C for 12 h [OSM, oven-dried sea cucumber meal]. After drying, each batch was milled to produce a meal, first with a coffee grinder (Krupps Spiver Grinder GX4100) and then with a mill (Ciclotec Tecator). Representative samples were taken for proximate and amino acid analysis and the remaining meal stored in sealed plastic bags at 4 °C until use.

The LSM had a very high salt content (>50 g/100 g), preventing its use in animal studies. It was therefore washed repeatedly in cold water and re-dried, constituting a different preparation treatment [LWSM, lyophilized washed sea cucumber meal].

### Diets

Isonitrogenous (120 g protein /kg) diets for rats were formulated according to [15] (Table 1). Lactalbumin was the sole protein source (120g protein/kg) in the control diets (CNC, negative control with no cholesterol; CC, positive control diet with 2% cholesterol). Sea cucumber meal was added to diets by substitution for lactalbumin, and accounted for half of dietary protein (60 g/kg) in the three experimental diets (LWS50, 50% protein from lyophilized-washed sea cucumber meal [LWSM]; CS50, 50% protein from cooked sea cucumber meal [CSM]; and OS50, 50% protein from oven-dried sea cucumber meal [OSM]). All diets were supplemented with methionine, lysine and tryptophan. Cholesterol (Sigma, Mexico) (20 g/kg) was added to the CC, LWS50, CS50 and OS50 diets. The CNC diet was cholesterol-free.

**Table 3:** Growth performance in rats fed diets containing sea cucumber meals during a 16-day experimental period.

	**Diets** ^2^				
**Parameters** ^1^	**CNC**	**CC**	**LWS50**	**CS50**	**OS50**
DM Intake *g*/d^3^	12.58±0.47^b^	13.06±0.37^ab^	12.60±24^b^	13.58+0.39^a^	12.45±0.59^b^
N Intake *g/d*	0.24±009^c^	0.29±0.001 ^a^	0.28±0.005^ab^	0.29±0.008^ab^	0.27±0.008 ^b^
Lipid intake *g/d*	0.69±0.02^c^	0.72±0.02 ^a^	0.69±0.01^ab^	0.74±0.02^ab^	0.68±0.03 ^b^
DM feces *g/d*	0.61±0.11^c^	1.12±0.3^b^	1.02±0.27^bc^	1.98±0.5^a^	1.14±0.1^b^
Wet weight gain *g/d* ^4^	4.20±0.23^a^	3.88±0.39^b^	3.64±1.6^bc^	3.17±0.23^bc^	2.54±0.71^c^
PER *g/g* ^5^	3.18±0.79^a^	2.54±0.21^ab^	2.33 ±0.92^ab^	1.95±0.31^b^	1.69±0.41^b^

^abc^Different letter superscripts in the same row indicate significant difference (p≤0.05) ^1^.Values = mean ± SD (n=4) ^2^.Diets: CNC = control with no added cholesterol; CC = Control with 1% cholesterol; LWS50 = 50% lyophilized washed sea cucumber + CC; CS50 = 50% cooked sea cucumber + CC; OS50 = 50% oven-cooked sea cucumber + CC ^3^.DM = Dry matter (grams per day) ^4^.Initial weight = 140±1135 g wet weight ^5^.Protein Efficiency Ratio = wet weight gain in grams / protein intake in grams.

### Experimental Animals

Male Wistar (Harlan strain) rats raised in the Animal Unit, Dr. Hideyo Noguchi Regional Research Center, were transferred to the animal facility at CINVESTAV-Merida and housed in standard cages during the adaptation and experimental periods. Temperature (24 ± 2 °C), photoperiod (12H light/dark) and relative humidity (65 ± 20%) were controlled. Animals had free access to water at all times. During the first three days of adaptation, all animals were fed a non-purified diet (Teklan Global Diets Rodents). For the remaining seven days of adaptation, five rats were fed only the CNC and the remaining rats were fed the CC.

### Experiment

Twenty rats (60 days old; initial weight = 140±11.35 g; CC diet during adaptation period) (five per treatment) were fed a control diet (CC) or an experimental diet (LWS50, CS50 or OS50) for a 16-day period (Table 1). Five rats (CNC during adaptation period) were fed the CNC diet. A fixed daily amount of feed was offered in two equal portions (approx. 150 g /kg body weight /d) twice daily at 09:00 and 18:00 h. Feed amount offered was based on daily intake of rats of a similar age fed a soy-based diet [16]. 

Experimental protocols were approved by the Institutional Animal Care and Use Committee of the Center for Research and Advanced Studies (Centro de Investigacion y de Estudios Avanzados del IPN) and comply with the applicable Mexican Official Norm (NOM-062-ZOO-1999), “Technical Specifications for the Care and Use of Laboratory Animals”, as well as all applicable federal and institutional regulations. 

### Lipid Analyses

After 16 days, the animals were fasted for 12 hours, and terminally anesthetized with ZOLETIL^®^ (tiletaminchlorhydrate/zolazepanchlorhydrate) via intramuscular injection (dose = 1mg/kg body weight). Blood samples were taken directly from the heart, killing the animal, and the liver removed immediately thereafter. After clotting, blood samples were centrifuged at 365 *g* for 20 min, the serum collected and total triglycerides and cholesterol determined with a COBAS C111 counter-top multi-analyzer (Roche, Mexico City). High density lipoproteins (HDL) and low density lipoproteins (LDL) were measured using enzymatic-colorimetric reactions [17]. 

Livers were removed and weighed. After taking samples for histological and gene expression analysis, the livers were snap frozen in liquid nitrogen and kept frozen at -70 °C until further analysis. Liver total lipids analysis was performed as described elsewhere [18], while triglycerides and cholesterol concentrations were analyzed according to [19]. 

Nitrogen content in the lyophilized carcass and feces samples was measured with a Flash EA1112 Analyzer. Lipid content was measured by extraction (1:100 w/v) with a chloroform/methanol (2:1 v/v) mixture as described elsewhere [20]. Amino acid analysis was done using a four-step Pico-Tag method (Waters, Corporation, Milford, MA, USA). Hydrolysis was carried out using 6 mol L^-1^ under vacuum at 104 °C for 24 h, followed by drying with an ethanol/water/triethylamine (2:2:1 v/v/v) solution and derivatization with ethanol/triethylamine/water/phenylisothiocyanate (7:1:1:1 v/v/v/v) reagent. Once derivatized, aliquots were subjected to reverse-phase high pressure liquid chromatography (HPLC).

### Fatty Acid composition

Fatty acid composition of sea cucumber meals and livers was analyzed by gas chromatography. Lipid extraction from the meals was done following [18] with some modifications. A 2:1 chloroform:methanol mixture was used and samples extracted for three days in darkness. After extraction, the suspensions were filtered and then dried in a N_2_ atmosphere. Saponification was done using KOH in 10% MeOH (50 mg:2 ml proportion)[21]at 80°C for 45 min. Fatty acids were recovered with three to four hexane washings and the saponified sample dried in a N_2_ atmosphere. After weighing, samples were derivatized according to [22] using BX3/CH3OH.Liver fatty acid composition was determined according to [22]. Briefly, 50 mg sample were mixed with 2 ml BX3/CH3OH and an internal standard (1 mg/10 mg fat, nonadecanoic acid, ME Supelco). The mixture was heated to 100 °C for 1 h and cooled to room temperature. Aliquots (1 ml hexane, 2 ml H_2_O) were added, and the mixture vortexed for 15 seconds. It was then centrifuged at 3000 rpm for 2 min and the fatty acid methyl esters (FAME) extracted from the upper hexane phase. The FAME were analyzed using an Agilent6890N gas chromatographer (Agilent, DF, Mexico) attached to a Agilent 5973 mass detector with a column (Supelco SPTM-2560, 100 m length, 0.25 mm internal diameter, 0.20 μm film thickness; Supelco, Mexico City). Runs were done in FULL SCAN mode. Helium was used as carrier gas at a 1 ml / min flow rate. Run conditions were: initial temperature, 140 °C x 5 min; 4 °C/min increases to 240 °C; 240 °C x 10 min. Peaks were identified by comparison with mass spectra in the NIST2011 database. The standard was a Supelco TM 37 (Component FAME Mix, Catalog No: 47885-U). Results were expressed as an average, as a percentage (%) for the sea cucumber meals and as mg/g sample in liver samples, using the average mg value from four replicates with a standard deviation.

### Histological Analysis

Liver samples were fixed with 10% formalin and treated with a tissue processor (AutotechniconDuo^®^). They were then introduced into paraffin blocks (Richard-Allan Scientific Paraffin Type 6^®^), and cut into two sections (2 µm thickness) with a microtome (Thermo Scientific Microm HM 325^®^). One section was dyed with hematoxilin-eosine stain and the other with periodic acid-Schiff stain to evaluate neutral and alkaline microsubstances. Unprocessed fragments were cut into 5 µm sections under freezing conditions using a cryostat, fixed in 10% formalin, and stained with Oil Red O. Sections were examined with a conventional optical microscope and images taken with a digital camera (Evolution™ LC Color). Presence of hepatic steatosis was evaluated semi-quantitatively according to [23].

### Gene Expression Analysis

Ribonucleic acid (RNA) was isolated from 100 mg liver from each organism with Trizol reagent (Invitrogen, USA), analyzed by 1% agarose gel electrophoresis, and quantified using a NanoDrop 2000c spectrophotometer (Thermo Scientific). RNA samples (5µg) were reversed transcribed using Improm II Reverse transcriptase (Promega) following the manufacturer protocol. Quantitative real time PCR (qRT-PCR) was done using Fast SYBR Green Master Mix (Applied Biosystems) following manufacturer instructions, and the StepOnePlus Real-Time PCR System (Applied Biosystems). Specific primer sequences for each gene are listed in table S1. Actin was used as housekeeping gene and relative gene expression was analyzed using the 2(-Delta Delta C(T)) method [24].

**Table 4:** Serum total triglycerides (Tg), total cholesterol (TC) and lipoproteins (LDL and HDL) levels, and the atherogenic index (AI) in rats fed a control or experimental diet containing sea cucumber (*I. badionotus*) meal.

	**Diets** ^2^				
**Parameters** ^1^	**CNC**	**CC**	**LWS50**	**CS50**	**OS50**
Tg *mg/dl*	38.19 ± 20.32^c^	83.98 ± 15.82ª	78.37 ± 22.43ª^b^	53.73 ± 18.70^b^	43.16 ± 10.57^c^
TC *mg/dl*	45.66 ± 6.58^d^	84.81 ± 10.0^a^	62.41 ± 5.0^bc^	55.19 ± 3.49^cd^	68.41 ± 6.52^b^
HDL *mg/dl*	50.94 ± 6.37^a^	50.54 ± 6.17ª	43.81 ±3.67ª^b^	41.38± 2.25^b^	44.62 ± 4.96ª^b^
LDL *mg/dl*	8.89 ± 3.98^d^	49.55 ± 9.40ª	23.17 ± 4.79^c^	20.89 ± 2.87^c^	33.11 ± 5.71^b^
AI^3^ *LDL/HDL*	0.17 ± 0.06 ^d^	0.98 ± 0.09 ª	0.52 ± 0.04 ^c^	0.50 ± 0.02 ^c^	0.74 ± 0.04 ^b^

1 Values = means (n= 4) ± SD, in milligrams / deciliter; ^abcd^Different letter superscripts in the same row indicate statistical difference (p<0.05) ^2^.CNC = control no added cholesterol; CC = Control with 1% cholesterol; LWS50 = 50% lyophilized washed sea cucumber + CC; CS50 = 50% cooked sea cucumber + CC; OS50 = 50% oven-cooked sea cucumber + CC ^3^;Atherogenic index.

**Table 5:** Liver total triglycerides (Tg), total cholesterol (TC) and total lipids (TL) levels in rats fed a control or experimental diet containing sea cucumber (*I. badionotus*) meal.

	**Diets** ^2^				
**Parameters** ^1^	**CNC**	**CC**	**LWS50**	**CS50**	**OS50**
Tg *mg/g dry liv*	46.81± 4.74^c^	89.97± 5.5^a^	60.22± 7.96^b^	47.59±6.17^c^	65.95±7.94^b^
TC *mg/g dry liv*	3.07 ± 0.23^b^	13.78 ± 4.29^a^	4.50 ± 1.25^b^	12.01 ± 1.75^a^	10.89 ± 1.7^a^
TL *mg/g dry liv*	177.36± 41.5^c^	328.61 ± 35^ab^	196.79± 17^c^	378.82±70^a^	272.31 ± 35^b^

1 Values = means (n= 4) ± SD, in milligrams / gram dry liver; ^abc^Different letter superscripts in the same row indicate statistical difference (p<0.05) ^2^.CNC = control no added cholesterol; CC = Control with 1% cholesterol; LWS50 = 50% lyophilized washed sea cucumber + CC; CS50 = 50% cooked sea cucumber + CC; OS50 = 50% oven-cooked sea cucumber + CC.

### Statistical analysis

A one-way ANOVA (Welch’s test) model was applied to analyze growth and metabolic variables: *Y*_*ij*_= µ + α_*i*_ +*ε*_*ij*_, where µ *=* population average; *α*_*i*_= treatment effect; and *ε*_*ij*_= random error. Differences between the means were identified with a Tukey test [25]. All analyses were performed using the Statgraphic statistics package (GraphPad Software Inc., San Diego, CA, USA)**.**

## Results and Discussion

### Sea cucumber meal composition

In general, the sea cucumber meals had high protein but low fat contents, although protein content varied by treatment (Table 2). Salt levels were high in all meals, but particularly so in the lyophilized meal (LSM). Dialysis of LSM had little effect on its salt content. However, extensive washing (LWSM) greatly reduced (p<0.05) salinity, as did thermal-water processing [cooking at 100 °C] (CSM). All sea cucumber meals were low in histidine, methionine, tryptophan and lysine (Table 2). They did however contain high levels of arginine and non-essential amino acids, such as glutamic acid, glycine and alanine. Fatty acid composition indicated that sea cucumber is rich in arachidonic acid, corroborating previous studies [26–29]. Heat treatment affected fatty acid composition. Meal composition was similar to that reported for other species such as *Holothuria forsakali* [30], *Cucumaria frondosa* [26] and *Stichopus japonicus* [31]. Its amino acid profile was better than reported for other species [27]. *Isostichopus badionotus* body wall was rich in collagen (data not shown), low in fat and very rich in polysaccharides. The meals’ lysine:arginine ratio was low (Table 1). Consumption of arginine-rich and low lysine:arginine ratio diets has been associated with beneficial effects on serum and liver cholesterol concentration [32,33].

**Table 6:** Fatty acid methyl ether (FAME) concentration in processed sea cucumber (*I. badionotus*) meals.

	**Meals** ^2^		
**Fatty acids** ^1^	**LWSM**	**CSM**	**OSM**
C14:0 (myristic)	5.49 ± 0.64^a^	5.26 ± 0.30 ^a^	1.11 ± 0.18^b^
C15:0	1.47 ± 0.17 ^a^	1.25 ± 0.04 ^b^	0.42± 0.05^c^
C16:0 (palmitic)	24.54 ± 1.30 ^a^	24.03± 0.64^a^	9.39 ± 0.69 ^b^
C16:1n-9 (palmitoleic)	13.75± 1.19 ^a^	14.23 ± 0.60 ^a^	4.28 ± 0.22 ^b^
C17:0	1.13± 0.63 ^a^	1.84 ± 0.02 ^a^	1.30 ± 0.21^a^
C17:1	1.89 ± 0.12 ^a^	1.06 ± 0.12 ^b^	0.80 ± 0.43 ^b^
C18:0 (stearic)	1.15 ± 0.65 ^c^	8.48 ± 0.40 ^a^	4.50 ± 0.15^b^
C18-1 (oleic)	10.04 ± 0.13^a^	4.45 ± 0.13 ^b^	2.20 ± 0.20 ^c^
C18:2 (linoleic)	4.85 ± 0.21 ^a^	3.59 ± 0.04 ^a^	5.91± 2.02 ^a^
C18:3	ND	ND	ND
C20:0	8.23 ± 0.41 ^b^	8.20 ± 0.42 ^b^	43.67 ± 0.08 ^a^
C20:1	1.44 ± 0.09 ^a^	1.55± 0.14 ^a^	1.51 ± 0.04 ^a^
C20:2	1.60 ± 0.14 ^a^	1.38 ± 0.26 ^a^	0.98 ± 0.09 ^b^
C20:3	3.41 ± 0.68 ^a^	3.25 ± 0.29 ^a^	3.09 ± 0.12 ^a^
C20:4 (arachidonic)	9.13 ± 0.066^b^	12.52± 0.005 ^a^	4.45 ± 0.36 ^c^
C20:5	1.45 ± 0.01 ^a^	1.73 ± 0.06 ^a^	0.43 ± 0.24^b^
C22:0	1.94 ± 0.22 ^a^	1.59 ± 0.12 ^b^	9.15 ± 0.40 ^a^
C22:1	2.60 ± 0.27 ^a^	2.30 ± 0.16 ^a^	2.52± 0.02 ^a^
C24:1	5.94± 0.77 ^a^	3.38± 0.33 ^b^	4.16 ± 0.18 ^b^
**Σ Fatty acids** ^3^	99.99	100.126	100.248
Σ SFA	43.95	50.62	69.59
Σ MUFA	35.63	26.98	15.49
Σ PUFA	20.41	22.52	15.16
Σ PUFA/ Σ SFA	0.46	0.445	0.22
Σ PUFA/ Σ MUFA	0.57	0.83	0.98

^1^ Concentration in g FAME/ 100 g sample; values are mean (n=3) ± standard deviation; ^abc^Different letter superscripts in the same row indicate statistical difference (p<0.05) ^2^.LWSM = lyophilized washed sea cucumber meal; CSM = water-cooked sea cucumber meal; OSM = oven-cooked sea cucumber meal. ND: not determined ^3^.SFA: saturated fatty acids; MUFA: monounsaturated fatty acids; PUFA: polyunsaturated fatty acids; ∑Fatty acids: ∑SFA + ∑MUFA +∑PUFA; ∑SFA = C14:0 + C16:0 + C18:0 + C20:0 21:0 + C22:0 + C23:0 + C24:0; ∑MUFA = C16:1 + C18:1n9t+ C24:1;∑PUFA: C18:2n6t + C20:4n6 + C22:2 + C20:5n3 + C22:5

**Table 7:** Fatty acid methyl ether (FAME) concentration in livers from rats fed control and experimental diets.

	**Diets** ^2^				
**Fatty acids** ^1^	**CNC**	**CC**	**LWS50**	**CS50**	**OS50**
C14:0	0.19± 0.05^c^	1.11±0.12 ^a^	0.23 ± 0.004^bc^	ND	0.33 ± 0.003^b^
C15:0	ND	ND	0.29± 0.09^a^	ND	0.32± 0.039^a^
C16:0	20.42 ± 3.4^d^	36.13 ± 3.13 ^a^	17.614± 6.88^cd^	33.0± 5.67^ab^	27.71 ± 6.43^bc^
C16:1n-9	1.70 ± 0.20^b^	10.35 ± 1.22^a^	4.08 ± 0.82^b^	8.68 ± 1.01^a^	10.43 ± 4.2^a^
C17:0	ND	ND	ND	ND	0.49± 0.091
C18:0	14.48 ± 2.85^a^	11.36 ± 2.79 ^a^	8.96 ± 2.36^c^	15.02 ± 1.2^ab^	12.24 ± 3.39^c^
C18:1n-9	11.39± 3.04^c^	47.43 ±10.56 ^b^	8.96 ± 2.36 ^d^	24.99 ± 5.19^b^	15.20 ± 7.8^c^
C18:2n-6	18.02 ±6.19^b^	33.49± 13.8 ^a^	18.04 ± 8.70 ^b^	46.15 ± 6.49^a^	41.59 ± 5.57^a^
C18:3n-6	ND	ND	0.278± 0.07	0.278± 0.07	1.18± 0.71
C20:0	ND	2.21± 0.50 ^a^	ND	ND	1.05± 0.71^b^
C20:1	ND	ND	ND	ND	0.25± 0.03^b^
C20:2	ND	ND	0.82± 0.3^a^	ND	0.87± 0.23^a^
C20:3	ND	ND	1.30± 0.06^b^	ND	2.33± 0.31 ^a^
C20:4n-3	8.96± 1.25^a^	7.04 ±0.05^a^	4.60± 0.71 ^b^	ND	7.53 ± 1.86^a^
C24:0	ND	ND	ND	ND	0.33± 0.03
C24:1	ND	ND	ND	ND	0.48± 0.11
C22:6n-3	2.37± 0.35^ab^	1.53±0.089^bc^	0.90± 0.17^c^	ND	2.09± 0.55^bc^
**Σ Fatty acids** ^3^	77.29	150.65	94.78	128.118	83.73
Σ SFA	35.09	51.57	24.55	61.88	44.43
Σ MUFA	13.09	58.17	18.81	62.38	30.62
Σ PUFA	31.22	39.41	23.07	55.68	23.98
Σ PUFA/ Σ SFA	0.88	0.76	0.93	0.89	0.54
Σ PUFA/ Σ MUFA	2.38	0.68	1.23	0.89	0.78

^1^ Concentracion in g FAME/100 g sample; values = mean (n= 4)± standard deviation; ^abcd^Different letter superscripts in the same row indicate statistical difference (p<0.05) ^2^.CNC = control no added cholesterol; CC = Control with 1% cholesterol; LWS50 = 50% lyophilized washed sea cucumber + CC; CS50 = 50% cooked sea cucumber + CC; OS50 = 50% oven-cooked sea cucumber + CC ^3^.SFA: saturated fatty acids; MUFA: monounsaturated fatty acids; PUFA: polyunsaturated fatty acids;∑Fatty acid = ∑SFA + ∑MUFA +∑PUFA; ∑SFA = C14:0 + C16:0 + C18:0 + C20:0 21:0 + C22:0 + C23:0 + C24:0; ∑MUFA = C16:1 + C18:1n9t + C20:1+ C24:1; ∑PUFA = C18:2n6t + C18:3n-6 + C20:3+ C20:4n6 + C22:6n3.

### Nutritional Parameters

**Figure 1:**
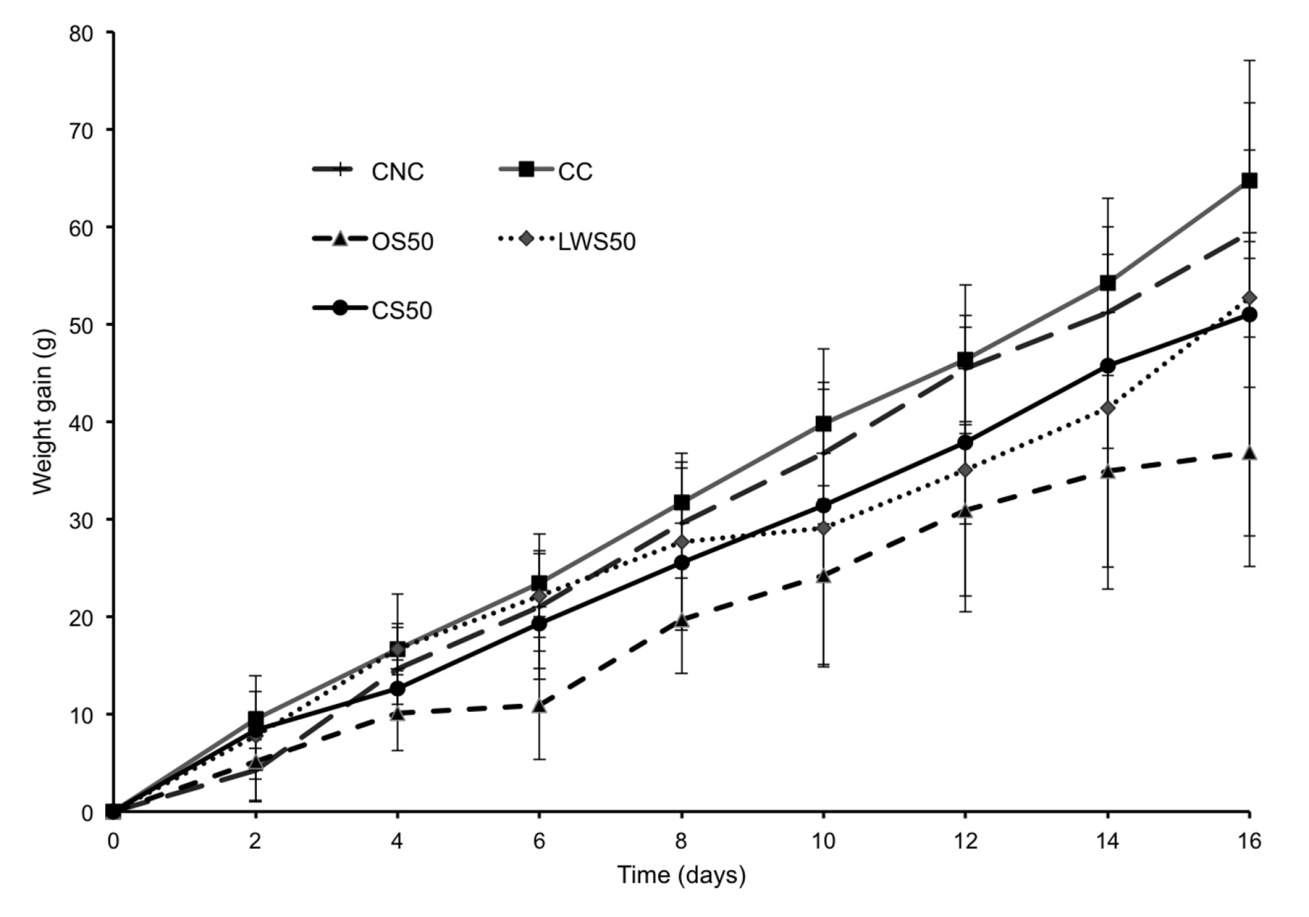
Body weight in rats during dietary supplementation period. Growth during a 16-day period in rats (63 ± 4 g initial weight) fed equivalent daily amounts of a lactalbumin control diet with no added cholesterol (CNC); a lactalbumin control diet with 2% added cholesterol (CC); a diet containing 50% protein from cooked sea cucumber meal (CS50); one containing 50% protein from oven-cooked sea cucumber (OS50); or one containing 50% protein from lyophilized washed sea cucumber meal (LWS50). Values are means ± SD, N = 5.

Sea cucumber is consumed as a functional food due to its putative health benefits. However, rigorously established conclusions about its nutritional and health-promoting properties *in vivo* are scarce. Diets containing 50% of their protein from sea cucumber *I. badionotus* meals [LWS50, CS50 and OS50] were readily consumed, and supported growth in young rats (Figure 1 and Table 3). However, the meals’ poor essential amino acids profile could not support growth at the same high rate as observed in the control rats (CNC and CC). As a result, protein efficiency ratio (PER) values were low. The poorest growth and PER values were produced with OS50. 

**Table 8:** Degree of steatosis in the livers of rats fed a control or experimental diet containing sea cucumber (*I. badionotus*) meal.

**Diets^1^**	**Normal** (%)	**Degree of Steatosis** ^2^			
		**< 5%**	**5-33%**	**33-6%**	**> 66%**
CNC	100	-	-	-	-
CC		40	-	20	40
LWS50	100	-	-	-	-
CS50	20	-	-	-	80
OS50	60	20	-	-	20

Values are the average of five replicates ^1^.CNC = control with no added cholesterol; CC = Control with 1% cholesterol; LWS50 = 50% lyophilized washed sea cucumber + CC; CS50 = 50% cooked sea cucumber + CC; OS50 = 50% oven-cooked sea cucumber + CC ^2^.Degree of steatosis diagnosis score: < 5%: minimal steatosis; 5- 33%: moderate steatosis; 33 - 66%: high steatosis. greater than 66%: severe steatosis [21].

### Serum and liver lipids

High lipid levels, particularly cholesterol associated with atherogenic lipoproteins, are a clear risk for CVD [34]. The influence of sea cucumber intake on lipid metabolism was evaluated using serum and liver lipids in rats fed sea cucumber or control diets supplemented with cholesterol (20 g/kg diet) (Table 1). Total triglycerides, total cholesterol, LDL-cholesterol and HDL-cholesterol were quantified in serum (Table 4), and total lipids, total triglycerides and total cholesterol measured in the liver (Table 5). Cholesterol supplementation in the control diet (CC) more than doubled total serum cholesterol in rats and had a similar effect on total triglycerides. This was also evident in the liver. Animals fed any one of the diets containing sea cucumber meal had lower levels of circulating cholesterol and cholesterol in the liver; these reductions were significant (p<0.05) in LWS50, but not so (p>0.05) in CS50 and OS50. In contrast, circulating triglycerides were reduced (p<0.05) by the CS50 and OS50 meals, but not by the LWS50. Liver triglycerides levels were reduced by all the sea cucumber diets. 

Both LDL-cholesterol and HDL-cholesterol were evaluated to determine if changes in total circulating cholesterol levels were due to specific changes in any cholesterol-containing lipoproteins. As described in other studies [14,35,36], the rats fed cholesterol-rich diets had greatly increased atherogenic LDL levels (Table 4). LDL levels were dramatically reduced by sea cucumber intake, particularly of the LWS50 and CS50 treatments. In contrast, sea cucumber intake had little effect on circulating HDL. This could be of considerable importance since HDL levels are recognized as a protective marker for CVD [37,38] due to their effects on reverse cholesterol transport [39,40], and a wide variety of other beneficial effects [41,42]. 

The blood atherogenic index (LDL/HDL) is a ratio used to indirectly assess CVD risk [43]. The LWS50 and CS50 treatments clearly reduced the atherogenic index (AI) while the OS50 was less effective. Overall, the sea cucumber diets had a beneficial effect on organism lipoprotein profile.

### Fatty Acid composition

The FAME analysis demonstrated that the LSM had a composition similar to other sea cucumber species and that heat treatment affected fatty acid composition (Table 6).Consumption of the cholesterol-containing diets was shown to increase liver fatty acid deposition, particularly that of palmitic acid, and saturated fatty acids (Table 7).The LWSM and OSM diets caused significant reductions in fatty acids while the CSM diet did not (p<0.05).

### Liver (histology and gene expression)

**Figure 2:**
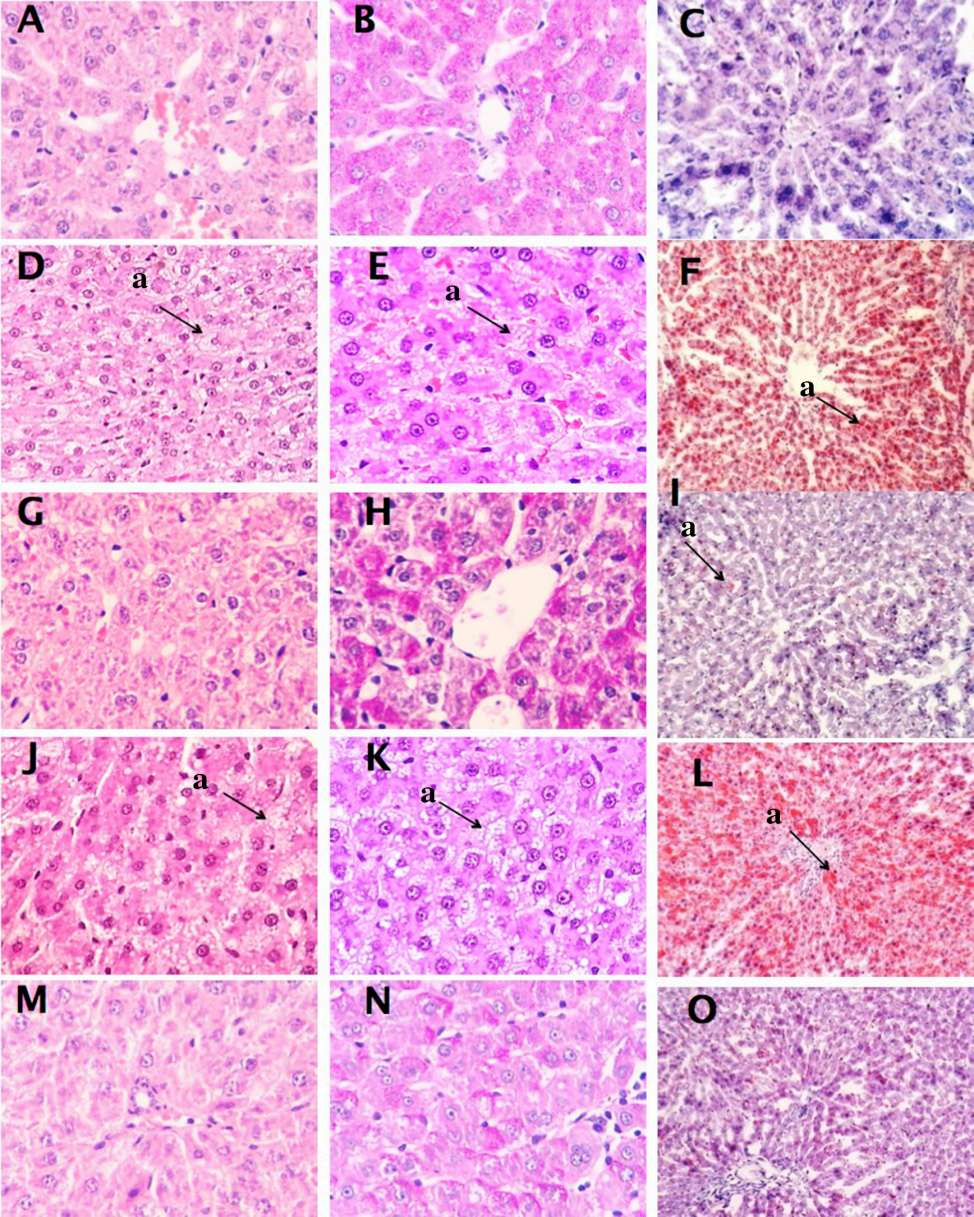
Photomicrographs (A-O) of livers from rats fed control cholesterol-free [CNC] or cholesterol supplemented [CC] diets or cholesterol supplemented experimental diets containing sea cucumber (*I. badionotus*) meal [LWS50, CS50, OS50] for 16 days. Sections A, D, G, J and M were stained with hematoxylin / eosin (H&E); B, E, H, K and N were stained with periodic acid-Schiff stain (PAS) and C, F, I, L and O were stained with Oil Red O. Magnification was 40X for H&E and PAS and 10X for Oil Red O. Consumption of sea cucumber limited the effects of dietary cholesterol on liver lipid deposition. CNC (A, B & C), no steatosis; CC (D, E & F), severe microvesicular steatosis; LWS50 (G. H & I), minor steatosis ; CS50 (J, K & L)  severe microvesicular steatosis ;  OS50 (M,N & O) minor steatosis with scattered lipid microvesicles.

Aberrant lipid accumulation in the liver elicits an inflammatory response in some individuals and can progress to non-alcoholic fatty liver disease and other hepatic disorders [44,45]. Histological analysis was done to determine if the observed changes in liver lipid levels had induced morphological changes in hepatocytes. Livers from the CNC treatment (no supplemental dietary cholesterol) exhibited normal hepatocyte staining with no appreciable triglyceride deposition (i.e. lipid droplets) in the cytoplasm (Figure 2, A, B and C). As expected, hepatocytes from rats given the cholesterol supplemented control diet (CC) had considerable deposition of microvesicular lipid droplets in the cytoplasm (Figure 2. D, E and F), which coincides with the triglyceride accumulation observed in the liver (Table 5). The LWS50 treatment caused a dramatic reduction in hepatocyte cytoplasm lipid levels and a total reduction in lipid droplets (Table 8; Figure 2, G, E and F); indeed, lipid levels were similar to those in livers from the CNC treatment (Figure 2, A and B). In contrast, the CS50 treatment resulted in considerable (100%) microvesicular steatosis (Table 8; Figure 2, J, K and L). The OS50 treatment had minimal effects on liver lipids (Figure 2, M, N and O).

**Figure 3:**
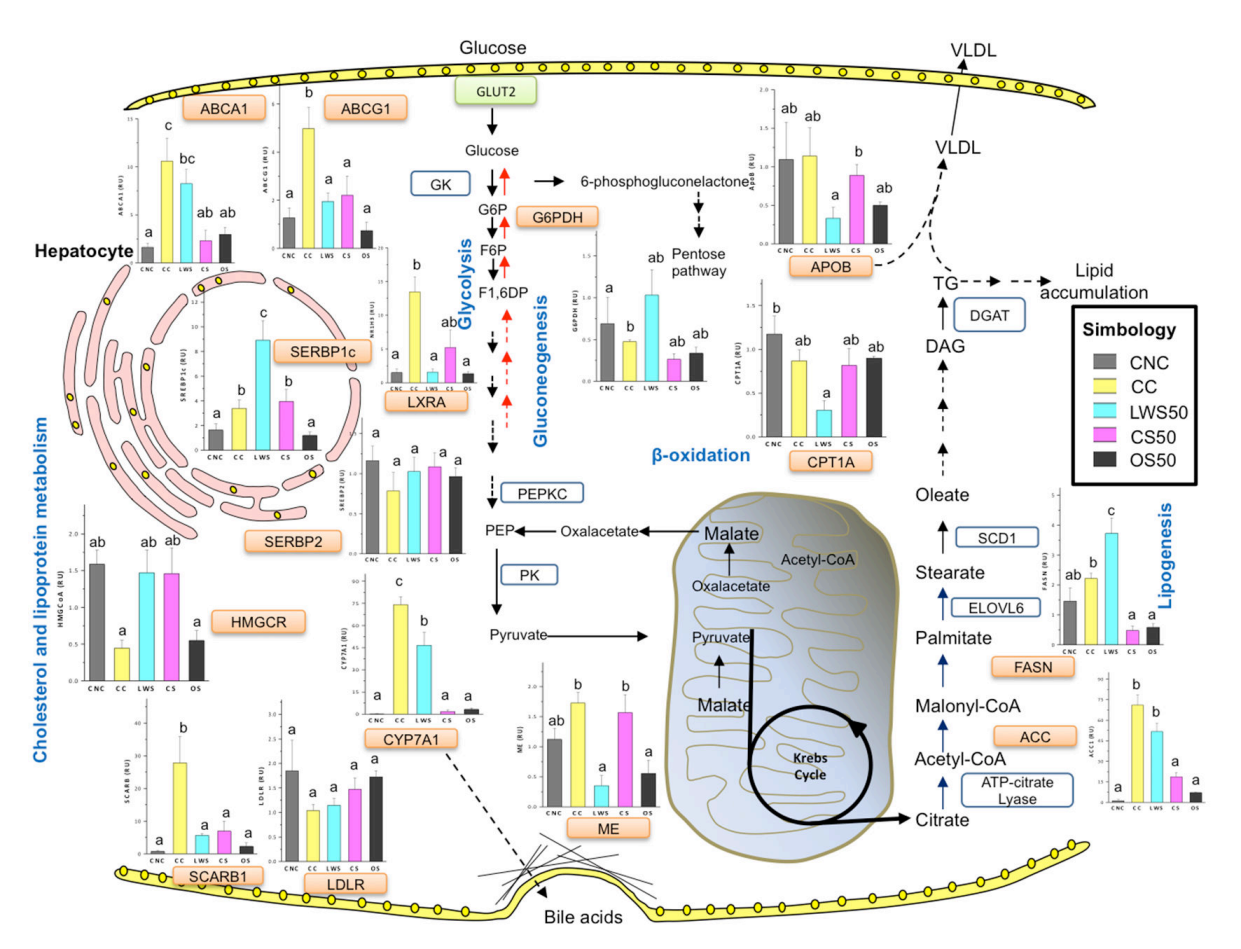
Effect of sea cucumber supplementation on expression of genes involved in lipid metabolism, a schematic representation. Rats were fed a lactalbumin control diet with no added cholesterol (CNC); a lactalbumin control diet with 2% added cholesterol (CC); a diet containing 50% protein from cooked sea cucumber meal (CS50); one containing 50% protein from oven-cooked sea cucumber (OS50); or one containing 50% protein from lyophilized washed sea cucumber meal (LWS50) for 16 days. Livers were collected to analyze mRNA expression. Gene expression was determined by RT-PCR analysis. Data were normalized to actin mRNA and fold-change are shown as the mean (n=5) ± SEM. Different letters within individual bar graphs indicate statistical difference (p<0.05). Schematically, increased cellular cholesterol levels leads to decreased cholesterol biosynthesis through the 3-Hydroxy-3-methylglutaryl CoA reductase (HMGCR) pathway and increased cholesterol efflux through the ATP-binding cassette transporter A1 and G1(ABCA1, ABCG1). In hepatocytes cholesterol elimination through the bile is mediated by Cholesterol 7 alpha-hydroxylase (CYP7A1). Excess of free cholesterol can be converted to their oxidized-derivatives the oxysterols, which are the natural ligands of the nuclear receptor Liver X receptor (LXR). LXR regulate the expression of ABCA1, ABCG1 and other enzymes involved in lipogenesis. High density lipoproteins (HDL) can be directly and selectively taken up by the liver via SCARB1. In hepatocytes, excess of lipids can be secreted via apolipoprotein-B (APOB)-containing lipoproteins (very low density lipoprotein [VLDL]). Triglycerides are synthesized from citrate through different enzymes including Acetyl-CoA carboxylase (ACC1) and fatty acid synthase (FASN) through the de novo fatty acid synthesis pathway. The carnitine acyltransferase (CROT) and carnitine palmitoyltransferase (CPT 1A) provide crucial steps in the transport of long fatty acids from the peroxisomes and to the mitochondria, respectively, regulating fatty acid oxidation. The peroxisome proliferator-activated receptors (PPARs) and their cofactors (PPARGC1A and PPARGC1B) can regulate different aspects of lipid and glucose metabolism. The sterol regulatory element binding proteins (SREBPs) can regulate cholesterol homeostasis (SREBP2) and fatty acid biosynthesis (SREBP1c).The carbohydrate-responsive element-binding protein (ChREBP) can regulate glycolysis and de novo fatty acid synthesis in the liver.

To evaluate the possible mechanism(s) behind the effects of sea cucumber meals on liver lipids, we assessed the mRNA expression of several key genes involved in cholesterol and lipid metabolism in liver. Expression of cholesterol 7-α-hydroxylase (CYP7A1), the rate-limiting enzyme in the classic pathway of bile acid biosynthesis for cholesterol elimination [46], was greatly increased in livers from the CC treatment (Figure 3). This increase in CYP7A1 expression exerted by cholesterol supplementation was deeply repressed in the CS50 and OS50 treatments, but only slightly affected in the LWS50. Oxysterols, which are cholesterol derivatives, activate transcription of several important lipid homeostasis pathways through the nuclear receptor liver X receptor alpha (LXRa, NR1H3 gene) [47]. Liver expression of LXRa was greatly enhanced in the CC treatment but curtailed in the sea cucumber treatments. LXRs induce expression of genes encoding proteins essential for cholesterol efflux and HDL biogenesis, including the ATP-binding cassette transporters A1 (ABCA1) and G1 (ABCG1) [48]. Rats in the CC treatment exhibited increased ABCA1 and ABCG1 expression in the liver. The opposite was true for ABCG1 expression in all the sea cucumber treatments and for ABCA1 expression in the CS50 and OS50 treatments. LXRs also stimulate lipogenesis in the liver through induction of sterol regulatory element-binding proteins (SREBP1c), acetyl CoA carboxylase (ACC1), fatty acid synthase (FASN) and stearoyl CoA desaturase1 (SCD1) [49,50]. The SREBPs transcription factors directly regulate expression of more than 30 genes involved in the synthesis and uptake of cholesterol, fatty acids, triglycerides and phospholipids [51]. The effects of high cholesterol diets on SREBP1c were partially abrogated only in the OS50 treatment. Induction of ACC1and FASN was reverted in the OS50 and CS50 treatments. As expected, expression of HMGCR (3-hydroxy-3-methyl-glutaryl-CoA reductase), the rate limiting enzyme in cholesterol biosynthesis, was reduced in the CC treatment. In contrast, no HMGCR reduction occurred in the sea cucumber treatments. The scavenger receptor class B member 1 (SR-BI, SCARB1 gene), known to facilitate cholesteryl ester uptake from HDL lipoproteins in the liver [52], increased in the CC treatment but not in the sea cucumber treatments.

**Figure 4:**
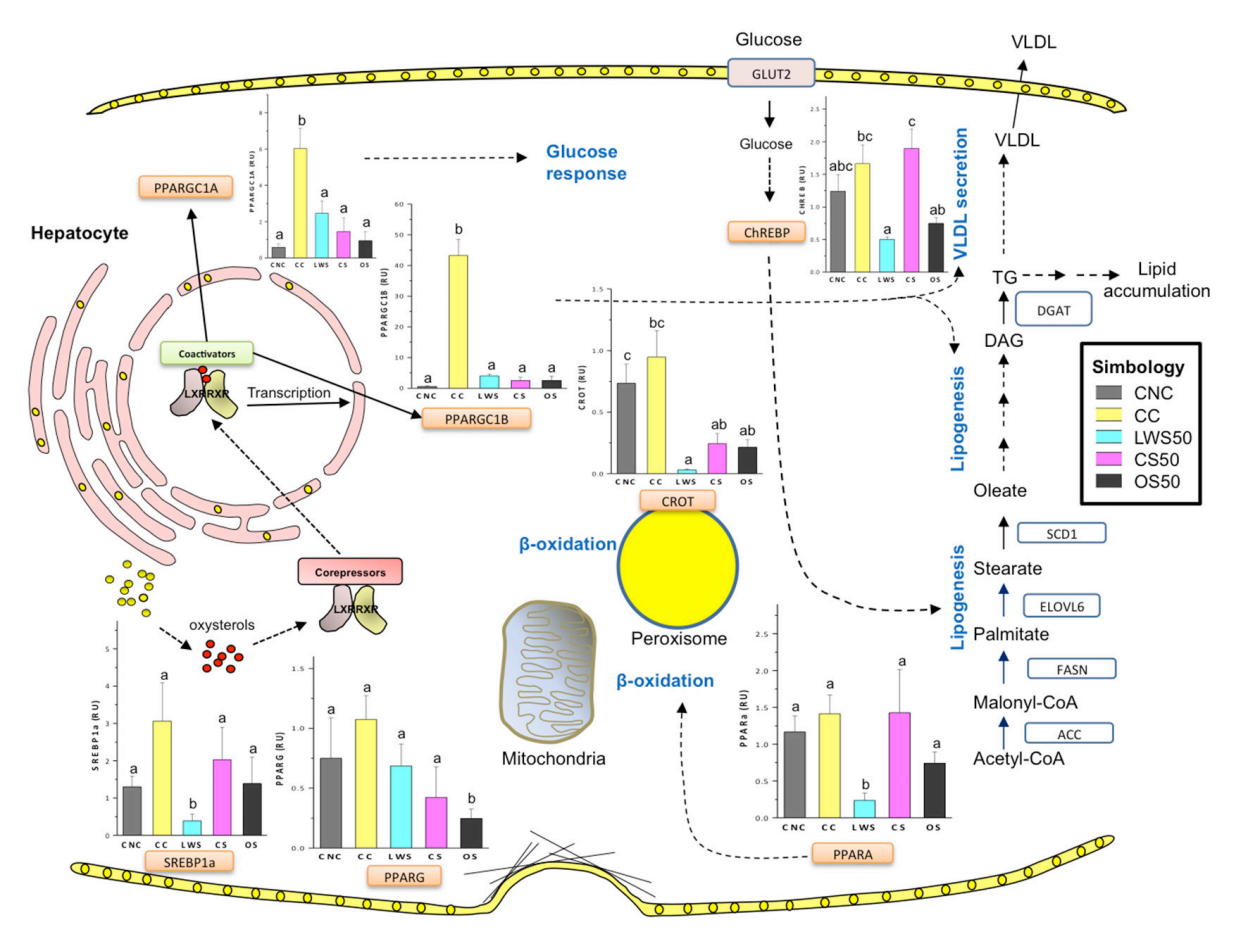
Effect of sea cucumber supplementation on expression of genes involved in lipid metabolism, a schematic representation. Rats were fed a lactalbumin control diet with no added cholesterol (CNC); a lactalbumin control diet with 2% added cholesterol (CC); a diet containing 50% protein from cooked sea cucumber meal (CS50); one containing 50% protein from oven-cooked sea cucumber (OS50); or one containing 50% protein from lyophilized washed sea cucumber meal (LWS50) for 16 days. Livers were collected to analyze mRNA expression. Gene expression was determined by RT-PCR analysis. Data were normalized to actin mRNA and fold-change are shown as the mean (n=5) ± SEM. Different letters within individual bar graphs indicate statistical difference (p<0.05). Schematically, increased cellular cholesterol levels leads to decreased cholesterol biosynthesis through the 3-Hydroxy-3-methylglutaryl CoA reductase (HMGCR) pathway and increased cholesterol efflux through the ATP-binding cassette transporter A1 and G1(ABCA1, ABCG1). In hepatocytes cholesterol elimination through the bile is mediated by Cholesterol 7 alpha-hydroxylase (CYP7A1). Excess of free cholesterol can be converted to their oxidized-derivatives the oxysterols, which are the natural ligands of the nuclear receptor Liver X receptor (LXR). LXR regulate the expression of ABCA1, ABCG1 and other enzymes involved in lipogenesis. High density lipoproteins (HDL) can be directly and selectively taken up by the liver via SCARB1. In hepatocytes, excess of lipids can be secreted via apolipoprotein-B (APOB)-containing lipoproteins (very low density lipoprotein [VLDL]). Triglycerides are synthesized from citrate through different enzymes including Acetyl-CoA carboxylase (ACC1) and fatty acid synthase (FASN) through the de novo fatty acid synthesis pathway. The carnitine acyltransferase (CROT) and carnitine palmitoyltransferase (CPT 1A) provide crucial steps in the transport of long fatty acids from the peroxisomes and to the mitochondria, respectively, regulating fatty acid oxidation. The peroxisome proliferator-activated receptors (PPARs) and their cofactors (PPARGC1A and PPARGC1B) can regulate different aspects of lipid and glucose metabolism. The sterol regulatory element binding proteins (SREBPs) can regulate cholesterol homeostasis (SREBP2) and fatty acid biosynthesis (SREBP1c).The carbohydrate-responsive element-binding protein (ChREBP) can regulate glycolysis and de novo fatty acid synthesis in the liver.

Nuclear receptor peroxisome proliferator-activated receptors (PPARs) are major carbohydrate and lipid metabolism regulators [53]. PPAR-α expression in liver was reduced in the LSW50 treatment and PPAR-γ was lower in the OS50 treatment (Figure 4). Peroxisome proliferator-activated receptor gamma co-activator 1-alpha (PGC-1α, PPARGC1A gene) regulates gluconeogenesis in the liver [54] and PGC1-beta (PGC-1β, PPRAGC1B gene) regulates fatty acid handling in the liver by co-activating the SREBPs [55]. Both were induced in the CC treatment but altered only minimally in livers from the sea cucumber treatments (Figure 4). Carnitine O-octanoyltransferase (CROT) and carnitinepalmitoyltransferase 1A (CPT 1A) encode key proteins involved in β-oxidation of fatty acids in the peroxisomes and mitochondria, respectively [56]. All the sea cucumber treatments reduced CROT expression in the liver (Figure 4) but only the LWS50 treatment reduced CPT 1A expression. 

In general, the changes in liver expression of genes involved in cholesterol and lipoprotein metabolism triggered by consumption of the high cholesterol control (CC) diet were abrogated by inclusion of sea cucumber meals in the diet.

The improved circulating and liver lipid profiles of rats fed diets containing LWS50 and CS50 may be due to a number of factors: 1) cholesterol elimination in the feces possibly facilitated by a conjugation reaction between bile acids and glycine [57,58]; 2) reduction in expression of the enzymes responsible for lipids biosynthesis [59]; 3) fatty acid β-oxidation may have increased [60]; and 4) levels of arginine, glycine and branching amino acids, associated with hypolipidic activity, were adequate [61]. In contrast to other studies [62], we found no evidence of increased cholesterol elimination through CYP7A1, SCARB1, ABCA1 or ABCG1. Moreover, we found no major changes in genes related to cholesterol biosynthesis (HMGCR), uptake (LDLR) or mitochondrial (CPT 1A) or peroxisomal (CROT) β-oxidation of fatty acids. However, we did find that induced expression (exerted by cholesterol supplementation) of genes contributing to the lipid biosynthesis pathway (NR1H3, FASN, ACC1A) and other co-factors (PPARGC1A and PPARGC1B) were clearly repressed in some of the sea cucumber treatments. This would explain the lower lipid levels in the liver, and probably in the plasma, when certain sea cucumber preparations were incorporated into the cholesterol-containing diets.

The presence of saponins in the sea cucumber preparations may have been involved in the observed effects. When added to experimental diets, CPT 1A was induced, β-oxidation of fatty acids increased and triglycerides were reduced [60]. This effect was mediated by an increase in carnitinepalmitoyltransferase activity in the liver and by induction of PPARa mRNA and their target genes [60]. Sea cucumber saponins are known to reduce dyslipidemia and liver lipid levels [63,64]. Sea cucumber is also rich in sulfated polysaccharides called glycosaminoglycans (GAGs), and sea cucumber GAGs are reported to reduce serum cholesterol in adult rats, although the mechanism remains unclear [14]. One study also indicates that glucosylceramide (GlcCer) from sea cucumber reduces both plasma and liver cholesterol levels in mice [62]. This may be partly due to increased LDL receptor expression, which would increase cholesterol uptake from plasma and increase its clearance. Revealing the degree to which saponins, GAGs and/or ClcCer contribute to the effects observed here will require extensive further research.

Understanding the biological effects of sea cucumber supplementation is vital to promoting their consumption as a functional ingredient. Using a dietary intervention and a variety of biochemical and molecular determinations, we have confirmed earlier reports of a hypolipidemic effect through diet supplementation with sea cucumber meal, probably due to bioactive substances (saponins or cerebrocides) contained within it [14,30,60]. Furthermore, we have shown that heat treatment may modify bioavailability of the bioactive compound(s) responsible for the beneficial effects of sea cucumber since only certain types of heat-treatment allowed the raw material to conserve its biological effects. These novel data clearly suggest that sea cucumber consumption may be beneficial for dyslipidemia, and confirm that the biological activity of substances in sea cucumber is clearly affected by preparation method.

## Conclusions

Sea cucumber *Isostichopus badionotus* from the coasts of Yucatan, Mexico, contains substances which reduce serum cholesterol and decrease LDL, thus improving the atherogenic index. A synergetic effect between amino acids profile, GAGs, altered bile acids production, and reductions in regulatory genes such as LXRA-SREBP1c axis and PPARs and their cofactors, may be the cause of the hypocholesterolemic effect observed here. These effects depend on the type of physicochemical treatment given the sea cucumber. Future research will need to focus on understanding the exact molecular mechanisms producing this effect. In particular, the nature of the responsible bioactive constituents needs to be defined since the present data suggest that some traditional processes commonly used to prepare sea cucumber products may greatly reduce or modify bioactive factors in the product, thereby negating any potential benefits.

## Supporting Information

Table S1 (DOCX)

## References

[1] Mathers CD, Loncar D (2006) Projections of global mortality and burden of disease from 2002 to 2030. PLOS Med 3(11): e442. doi:10.1371/journal.pmed.0030442. PubMed: 17132052. 17132052 PMC1664601

[2] Roger VL, Go AS, Lloyd-Jones DM, Benjamin EJ, Berry JD, et al. on behalf of the American Heart Association Statistics Committee and Stroke Statistics Subcommittee (2012) Heart disease and stroke statistics-2012 update: a report from the American Heart Association. Circulation 125(1): e2-e220. doi:10.1161/CIR.0b013e31823ac046. PubMed: 22179539. 22179539 PMC4440543

[3] World Health Organization (2011) Global Atlas on Cardiovascular Disease Prevention and Control. Mendis S Puska P Norrving B Geneva: World Health Organization p. 156.

[4] Cohen JC, Horton JD, Hobbs HH (2011) Human Fatty Liver Disease: Old Questions, New Insights. Science 332(6037): 1519-1523. doi:10.1126/science.1204265. PubMed: 21700865. 21700865 PMC3229276

[5] Odegaard AO, Koh WP, Gross MD, Yuan JM, Pereira MA (2011) Combined lifestyle factors and cardiovascular disease mortality in Chinese men and women: the Singapore Chinese health study. Circulation 124(25): 2847-2854. doi:10.1161/CIRCULATIONAHA.111.048843. PubMed: 22104554. 22104554 PMC3400937

[6] Liu K, Daviglus ML, Loria CM, Colangelo LA, Spring B et al. DM (2012) Healthy Lifestyle Through Young Adulthood and the Presence of Low Cardiovascular Disease Risk Profile in Middle Age: Clinical Perspective. The Coronary Artery Risk Development in (Young) Adults (CARDIA) Study. Circulation 125(8): 996-1004. doi:10.1161/CIRCULATIONAHA.111.060681. PubMed: 22291127. 22291127 PMC3353808

[7] Kelly MS (2005) Echinoderms: Their Culture and Bioactive Compounds. In Matranga V Echinodermata. Berlin: Springer-Verlag pp. 139-165. 17152697

[8] Conan DC, Sloan NA (1988) World Fisheries for Echinoderms. In Caddy JF Marine Invertebrate Fisheries: Their Assessment and Management. New York: John Wiley & Sons pp. 647-663.

[9] Zetina Moguel C, Rios Lara V, Koyoc Cruz M, Hernandez Herrera I, Cervera Cervera K et al. (2003) Sea cucumber (*Astichopus* *multifidus,* *Isostichopus* *badionotus* and *Holothuria* *floridana*) Biomass Estimation in Two Areas of Yucatan Coast between October of 2000 to March of 2001. Gulf Publishing House and Caribbean Fisheries Institute 54 pp. 298-306.

[10] Sugawara T, Zaima N, Yamamoto A, Noguchi R, Hirata T (2006) Isolation of sphingoid bases of sea cucumber cerebrosides and their cytotoxicity against human colon cancer cells. Biosci Biotechnol Biochem 70(12): 2906-2912. doi:10.1271/bbb.60318. PubMed: 17151482. 17151482

[11] Hawa I, Zulaikah M, Jamaludin M, ZainalAbidin AA, Kaswandi MA et al. (1999) The potential of the coelomic fluid in sea cucumber as an antioxidant. Mal. J Nutr 5: 55-59. 22692358

[12] Komano H, Mizuno D, Natori S (1980) Purification of lectin induced in the hemolyph of *Sarcophaga* *peregrina* larvae on injury. J Biol Chem 225: 2919-2924. 6766942

[13] Mojica ER, Merca FE (2005) Isolation and partial characterization of a lectin from the internal organs of sea cucumber (*Holothuria* *scabra* Jaeger). Inter J Zool Res 1(1): 59-65. doi:10.3923/ijzr.2005.59.65.

[14] Liu HH, Ko WC, Hu ML (2002) Hypolipidemic Effect of Glycosaminoglycans from the Sea Cucumber *Metriatyla* *scabra* in Rats Fed a Cholesterol-Supplemented Diet. J Agric Food Chem 50(12): 3602-3606. doi:10.1021/jf020070k. PubMed: 12033836. 12033836

[15] Olivera L, Rodriguez CR, Pereira PF, Cockburn J, Soldani FN et al. (2003) Nutritional and Physiological Responses of Young Growing Rats to Diets Containing Raw Cowpea Seed Meal, Protein Isolate (Globulins), or Starch. J Agric Food Chem 51: 319-325. doi:10.1021/jf0257749. PubMed: 12502427. 12502427

[16] Grant G, Dorward PM, Pusztai A (1993) Pancreatic enlargement is evident in rats fed diets containing raw soybeans (*Glycine* *max*) or cowpeas (*Vigna* *unguiculata*) for up to 800 days but not in those fed diets based on kidney beans (*Phaseolus* *vulgaris*), or lupinseed (*Lupinus* *angustifolius*). J Nutr 123: 2207-2215. PubMed: 7505319. 7505319 10.1093/jn/123.12.2207

[17] Diagnostics Roche (2009) Cholesterol Gen. 2.2009-12, v. 6. Mannheim: Roche Diagnostics GmbH.

[18] Folch J, Lees M, Sloane-Stanley GH (1957) A simple method for the isolation and purification of total lipids from animal tissue. J Biol Chem 226(1): 497-509. PubMed: 13428781. 13428781

[19] Lopes-Virella MF, Stone P, Ellis S, Colwell JA (1977) Cholesterol determination in high-density lipoproteins separated by three different methods. Clin Chem 23: 882–884. PubMed: 192488. 192488

[20] Waters Corporation (1984) Pico-Tag Amino Acid Analysis System Owner’s Manual. Milford, MA: Waters Corporation.

[21] Damiani MC, Cecilia A, Popovich CA, Constenla D, Patricia I, et al. (2010) Lipid analysis in *Haematococcus* *pluvialis* to assess its potential use as a biodiesel feedstock. Bioresour Technol 101: 3801–3807. doi:10.1016/j.biortech.2009.12.136. PubMed: 20117928. 20117928

[22] Araujo P, Nguyen TT, Frøyland L, Wang J, Kang XJ (2008) Evaluation of a rapid method for the quantitative analysis of fatty acids in various matrices. NHI Public Access. J Cromatography. Author Manuscript; 1212 (1-2): 106-113. PubMed: 18937951. 10.1016/j.chroma.2008.10.006 PMC2593112 18937951

[23] Kleiner DE, Brunt EM, Van Natta M, Behling C, Contos MJ et al. (2005) Design and validation of a histological scoring system for nonalcoholic fatty liver disease. Hepatology 41: 1313-1321. doi:10.1002/hep.20701. PubMed: 15915461. 15915461

[24] Livak KJ, Schmittgen TD (2001) Analysis of relative gene expression data using real-time quantitative PCR and the 2(-Delta Delta C(T)) Method. Methods 25(4): 402-408. doi:10.1006/meth.2001.1262. PubMed: 11846609. 11846609

[25] Sokal RR, Rohlf FJ (1995) The Principle and Practice of Statistics in Biological Research. 3rd edition. New York: WH Freeman and Co.

[26] Zhong Y, Khan MA, Shahidi F (2007) Compositional Characteristics and Antioxidant Properties of Fresh and Processed Sea Cucumber (*Cucumaria* *frondosa*). J Agric Food Chem 55: 1188-1192. doi:10.1021/jf063085h. PubMed: 17243707. 17243707

[27] Wen J, Hu Ch, Fan S (2010) Chemical composition and nutritional quality of sea cucumbers. J Sci Food Agric 90(14): 2469-3474. doi:10.1002/JSFA.4108. PubMed: 20718029. 20718029

[28] Yahyavi M, Afkhami M, Javadi A, Ehsanpour M, Khazaali A et al. (2012) Fatty acid composition in two sea cucumber species, *Holothuria* *scabra* and *Holothuria* *leucospilata* from Qeshm Island (Persian Gulf). Afr J Biotechnol Vol. 11(12): 2668-2668, February

[29] Hasegawa N, Sawaguchi S, Tokuda M, Unuma T (2013) Fatty acid composition in sea cucumber *Apostichopus* *japonicus* fed with microbially degraded dietary sources. Aquaculture Research. Article first published online: 26 FEB 2013. DOI: 10.1111/are.12149

[30] Rodríguez ME, González BC, Lamas M, Taboada C (2000) Nutritional value of *Holothuria* *forskali* protein and effects on serum lipid profile in rats. J Physiol Biochem 56(1): 39-44. doi:10.1007/BF03179775. PubMed: 10879680. 10879680

[31] Duan X, Zhang M, Mujumdar AS, Wang S (2010) Microwave freeze-drying of sea cucumber (*Stichopus* *japonicus*). J Food Eng 96: 491–497. doi:10.1016/j.jfoodeng.2009.08.031.

[32] Rajamohan T, Kurup PA (1997) Lysine: arginine ratio of a protein influences cholesterol metabolism. Part 1 - Studies on sesame protein having low lysine: arginine ratio. Indian J Exp Biol 35(11): 1218-1223. PubMed: 9567754. 9567754

[33] Ayo J, Carballo J, Serrano J, Olmedilla-Alonso B, Ruiz-Capillas C et al. (2007) Effect of total replacement of pork backfat with walnut on the nutritional profile of frankfurters. Meat Sci 77(2): 173-181. doi:10.1016/j.meatsci.2007.02.026. PubMed: 22061588. 22061588

[34] Tyroler HA (1984) Cholesterol and cardiovascular disease. An overview of Lipid Research Clinics (LRC) epidemiologic studies as background for the LRC Coronary Primary Prevention Trial. Am J Cardiol 54(5): 14C-19C. doi:10.1016/0002-9149(84)90851-8. PubMed: 6382998. 6382998

[35] Najwa S, Aljawad E, Fryer B , Fryer H (1991) Effects of casein, soy and whey protein and amino acid supplementation on cholesterol metabolism in rats. J Nutr Biochem 2 (3): 150-158. doi:10.1016/0955-2863(91)90007-R.

[36] Leontowicz H, Gorinstein S, Lojek A, Leontowicz M, Čı ´ ž M et al. (2002) Comparative content of some bioactive compounds in apples, peaches and pears and their influence on lipids and antioxidant capacity in rats. J Nutr Biochem 13(10): 603-610. doi:10.1016/S0955-2863(02)00206-1. PubMed: 12550072. 12550072

[37] Miller GJ, Miller NE (1975) Plasma-high-density-lipoprotein concentration and development of ischaemic heart-disease. Lancet 1(7897): 16-19. PubMed: 46338. 46338 10.1016/s0140-6736(75)92376-4

[38] Gordon DJ, Probstfield JL, Garrison RJ, Neaton JD, Castelli WP et al. (1989) High-density lipoprotein cholesterol and cardiovascular disease. Four prospective American studies. Circulation 79(1): 8-15. doi:10.1161/01.CIR.79.1.8. PubMed: 2642759. 2642759

[39] Miller NE, La Ville A, Crook D (1985) Direct evidence that reverse cholesterol transport is mediated by high-density lipoprotein in rabbit. Nature 314(6006): 109-111. doi:10.1038/314109a0. PubMed: 3974712. 3974712

[40] Khera AV, Cuchel M, Llera-Moya M, Rodrigues A, Burke MF et al. (2011) Cholesterol efflux capacity, high-density lipoprotein function, and atherosclerosis. N Engl J Med 364(2): 127-135. doi:10.1056/NEJMoa1001689. PubMed: 21226578. 21226578 PMC3030449

[41] Heinecke JW (2010) The protein cargo of HDL: implications for vascular wall biology and therapeutics. J Clin Lipidol 14(5): 371-375. PubMed: 20975842. 10.1016/j.jacl.2010.08.005 PMC2959168 20975842

[42] Säemann MD, Poglitsch M, Kopecky C, Haidinger M, Hörl WH et al. (2010) The versatility of HDL: a crucial anti-inflammatory regulator. Eur J Clin Invest 40(12): 1131-1143. doi:10.1111/j.1365-2362.2010.02361.x. PubMed: 20695882. 20695882

[43] Mertz DP (1980) Atherosclerosis-index (LDL/HDL): risk indicator in lipid metabolism disorders. Med Klin 75(4): 159-161. PubMed: 7374617. 7374617

[44] Cohen JC, Horton JD, Hobbs HH (2011) Human fatty liver disease: old questions and new insights. Science 332(6037): 1519-1523. doi:10.1126/science.1204265. PubMed: 21700865. 21700865 PMC3229276

[45] Greenberg AS, Coleman RA, Kraemer FB, McManaman JL, Obin MS et al. (2011) The role of lipid droplets in metabolic disease in rodents and humans. J Clin Invest 121(6): 2102-2110. doi:10.1172/JCI46069. PubMed: 21633178. 21633178 PMC3104768

[46] Chiang JY (2009) Bile acids: regulation of synthesis. J Lipid Res 50(10): 1955-1966. doi:10.1194/jlr.R900010-JLR200. PubMed: 19346330. 19346330 PMC2739756

[47] Janowski BA, Willy PJ, Devi TR, Falck JR, Mangelsdorf DJ (1996) An oxysterol signalling pathway mediated by the nuclear receptor LXR alpha. Nature 383(6602): 728-731. doi:10.1038/383728a0. PubMed: 8878485. 8878485

[48] Venkateswaran A, Laffitte BA, Joseph SB, Mak PA, Wilpitz DC et al. (2000) Control of cellular cholesterol efflux by the nuclear oxysterol receptor LXR alpha. Proc Natl Acad Sci U_S_A 97(22): 12097-12102. doi:10.1073/pnas.200367697. PubMed: 11035776. 11035776 PMC17300

[49] Repa JJ, Liang G, Ou J, Bashmakov Y, Lobaccaro JM et al. (2000) Regulation of mouse sterol regulatory element-binding protein-1c gene (SREBP-1c) by oxysterol receptors, LXRalpha and LXRbeta. Genes Dev 14(22): 2819-2830. doi:10.1101/gad.844900. PubMed: 11090130. 11090130 PMC317055

[50] Schultz JR, Tu H, Luk A, Repa JJ, Medina JC et al. (2000) Role of LXRs in control of lipogenesis. Genes Dev 14(22): 2831-2838. doi:10.1101/gad.850400. PubMed: 11090131. 11090131 PMC317060

[51] Horton JD, Goldstein JL, Brown MS (2002) SREBPs: activators of the complete program of cholesterol and fatty acid synthesis in the liver. J Clin Invest 109(9): 1125-1131. doi:10.1172/JCI200215593. PubMed: 11994399. 11994399 PMC150968

[52] Acton S, Rigotti A, Landschulz KT, Xu S, Hobbs HH et al. (1996) Identification of scavenger receptor SR-BI as a high density lipoprotein receptor. Science 271(5248): 518-520. doi:10.1126/science.271.5248.518. PubMed: 8560269. 8560269

[53] Semple RK, Chatterjee VKK, O’Rahilly S (2006) PPARgamma and human metabolic disease. J Clin Invest 116(3): 581–589. doi:10.1172/JCI28003. PubMed: 16511590. 16511590 PMC1386124

[54] Yoon JC, Puigserver P, Chen G, Donovan J, Wu Z et al. (2001) Control of hepatic gluconeogenesis through the transcriptional coactivator PGC-1. Nature 413(6852): 131-138. doi:10.1038/35093050. PubMed: 11557972. 11557972

[55] Lin J, Yang R, Tarr PT, Wu PH, Handschin C et al. (2005) Hyperlipidemic effects of dietary saturated fats mediated through PGC-1beta coactivation of SREBP. Cell 120(2): 261-273. doi:10.1016/j.cell.2004.11.043. PubMed: 15680331. 15680331

[56] Van der Leij FR, Huijkman NC, Boomsma C, Kuipers JR, Bartelds B (2000) Genomics of the human carnitine acyltransferase genes. Mol Genet Metab 71(1-2): 139-153. doi:10.1006/mgme.2000.3055. PubMed: 11001805. 11001805

[57] Lefebvre P, Cariou B, Lien F, Kuipers F, Staels B (2009) Role of Bile Acids and Bile Acid Receptors in Metabolic Regulation. Physiol Rev 89: 147–191. doi:10.1152/physrev.00010.2008. PubMed: 19126757. 19126757

[58] Herrmann RG (1959) Effect of taurine, glycine and β-sitoesterols on serum and tissue cholesterol in the rat and rabbit. Circ Res 7: 224-227. doi:10.1161/01.RES.7.2.224. PubMed: 13629821. 13629821

[59] Torres N, Torre-Villalvazo I, Tovar AR (2006) Regulation of lipid metabolism by soy protein and its implication in diseases mediated by lipid disorders. J Nutr Biochem 17(6): 365-373. doi:10.1016/j.jnutbio.2005.11.005. PubMed: 16481155. 16481155

[60] Hu XQ, Wang YM, Wang JF, Xue Y, Li ZJ et al. (2010) Dietary saponins of sea cucumber alleviate orotic acid-induced fatty liver in rats via PPARalpha and SREBP-1c signaling. Lipids Health Dis 9: 25. doi:10.1186/1476-511X-9-25. PubMed: 20211032. 20211032 PMC2846940

[61] Park T, Jooyeon Oh MS, Lee Kyungshin (1999) Dietary taurine or glycine supplementation reduces plasma and liver cholesterol and triglyceride concentrations in rats fed a cholesterol-free diet. Nutr Res 19(12): 1777-1789. doi:10.1016/S0271-5317(99)00118-9.

[62] Hossain Z, Sugawara T, Aida K, Hirata T (2011) Effect of dietary glucosylceramide from sea cucumber on plasma and liver lipids in cholesterol-fed mice. Fish Sci 77(6): 1081-1085. doi:10.1007/s12562-011-0407-y.

[63] Hu X, Li Z, Xue Y, Xu J, Xue C et al. (2012) Dietary saponins of sea cucumber ameliorate obesity, hepatic steatosis, and glucose intolerance in high-fat diet-fed mice. J Med Food 15(10): 909-916. doi:10.1089/jmf.2011.2042. PubMed: 22897583. 22897583

[64] Hu XQ, Xu J, Xue Y, Li ZJ, Wang JF et al. (2012) Effects of bioactive components of sea cucumber on the serum, liver lipid profile and lipid absorption. Biosci Biotechnol Biochem 76(12): 2214-2218. doi:10.1271/bbb.120495. PubMed: 23221720. 23221720

