# Correction: Diets Containing Sea Cucumber (*Isostichopus badionotus*) Meals Are Hypocholesterolemic in Young Rats

**DOI:** 10.1371/journal.pone.0118525

**Published:** 2015-03-06

**Authors:** The PLOS ONE Staff

There are errors in the title and legend of Fig. 1. The title should read “Weight gain in rats during dietary supplementation period.” In the legend, “(63 ± 4 g initial weight)” should read “(140 ± 11.35 g initial weight).” Please view the correct Fig. 1 title and legend here.

**Fig 1:**
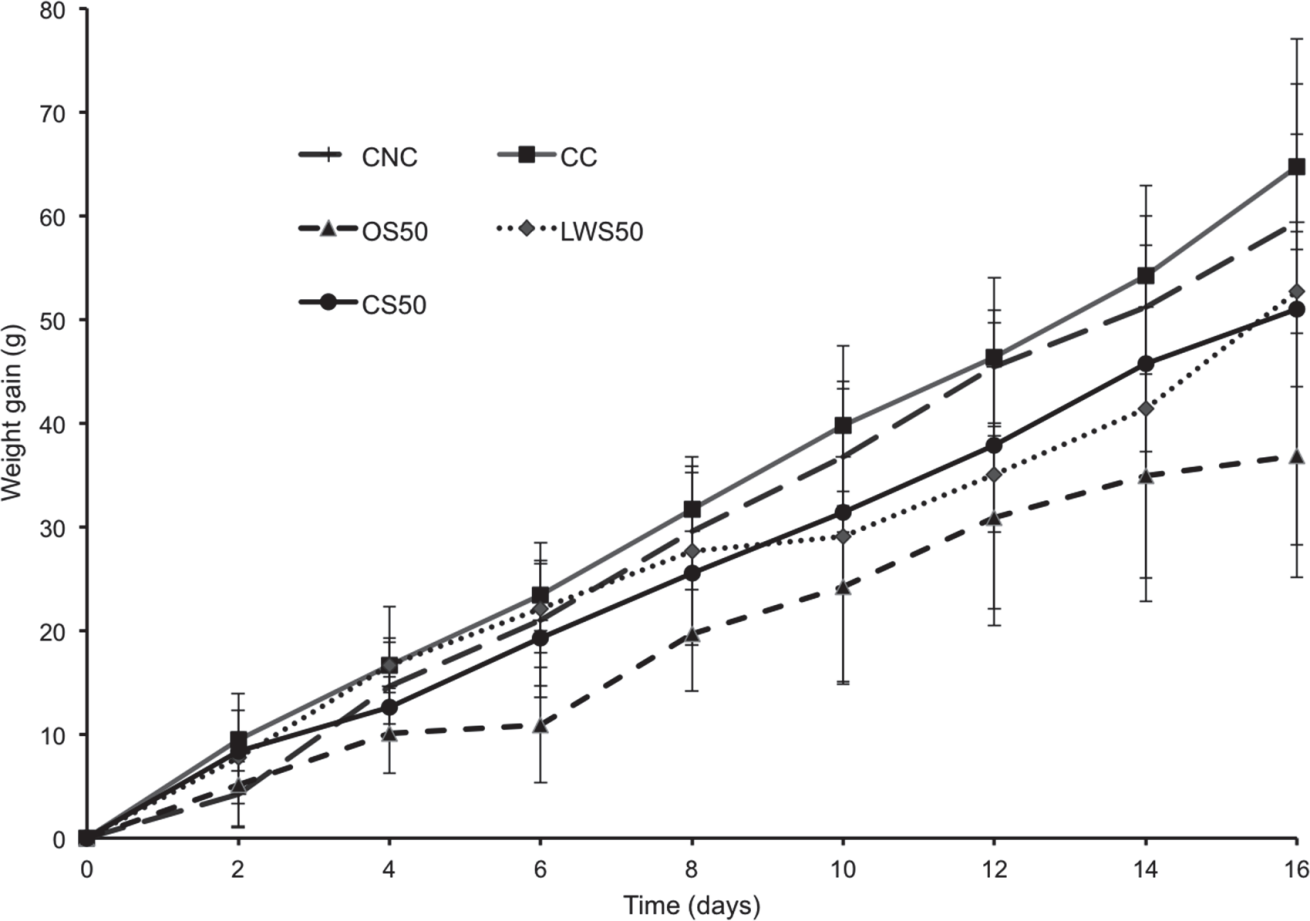
Weight gain in rats during dietary supplementation period. Growth during a 16-day period in rats (140 ± 11.35 g initial weight) fed equivalent daily amounts of a lactalbumin control diet with no added cholesterol (CNC); a lactalbumin control diet with 2% added cholesterol (CC); a diet containing 50% protein from cooked sea cucumber meal (CS50); one containing 50% protein from oven-cooked sea cucumber (OS50); or one containing 50% protein from lyophilized washed sea cucumber meal (LWS50). Values are means ± SD, N = 5.

Additionally, the following information is missing in the legend for Fig. 2: Lowercase “a” with an arrow indicates example of steatosis. Please view the complete, correct Fig. 2 legend here.

**Fig 2:**
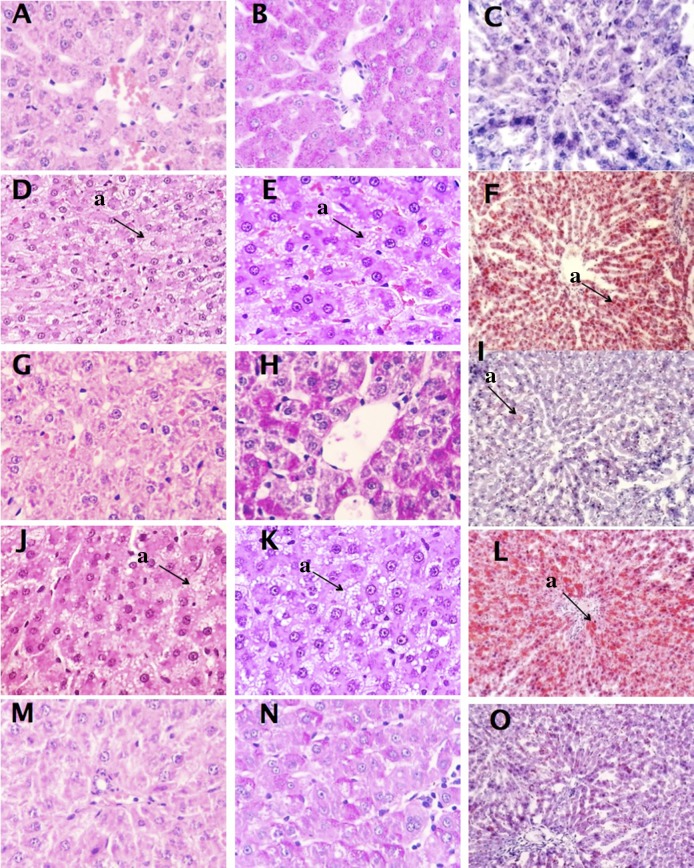
Photomicrographs (A-O) of livers from rats fed control cholesterol-free [CNC] or cholesterol supplemented [CC] diets or cholesterol supplemented experimental diets containing sea cucumber (*I*. *badionotus*) meal [LWS50, CS50, OS50] for 16 days. Sections A, D, G, J and M were stained with hematoxylin / eosin (H&E); B, E, H, K and N were stained with periodic acid-Schiff stain (PAS) and C, F, I, L and O were stained with Oil Red O. Magnification was 40X for H&E and PAS and 10X for Oil Red O. Consumption of sea cucumber limited the effects of dietary cholesterol on liver lipid deposition. CNC (A, B & C), no steatosis; CC (D, E & F), severe microvesicular steatosis; LWS50 (G. H & I), minor steatosis; CS50 (J, K & L) severe microvesicular steatosis; OS50 (M,N & O) minor steatosis with scattered lipid microvesicles. Lowercase "a" with an arrow indicates example of steatosis.

There are two errors in the footnotes for Table 3. For footnote 2, “CC = Control with 1% cholesterol” should read “CC = Control with 2% cholesterol.” For footnote 4, “Initial weight = 140±1135 g wet weight” should read “Initial weight = 140±11.35 g wet weight.” Please view the corrected Table 3 here.

**Table 3:** Growth performance in rats fed diets containing sea cucumber meals during a 16-day experimental period.

	Diets^2^				
Parameters^1^	CNC	CC	LWS50	CS50	OS50
DM Intake *g/d* ^3^	12.58±0.47^b^	13.06±0.37^ab^	12.60±24^b^	13.58+0.39^a^	12.45±0.59^b^
N Intake *g/d*	0.24±009^c^	0.29±0.001 ^a^	0.28±0.005^ab^	0.29±0.008^ab^	0.27±0.008 ^b^
Lipid intake *g/d*	0.69±0.02^c^	0.72±0.02 ^a^	0.69±0.01^ab^	0.74±0.02^ab^	0.68±0.03 ^b^
DM feces *g/d*	0.61±0.11^c^	1.12±0.3^b^	1.02±0.27^bc^	1.98±0.5^a^	1.14±0.1^b^
Wet weight gain *g/d* ^4^	4.20±0.23^a^	3.88±0.39^b^	3.64±1.6^bc^	3.17±0.23^bc^	2.54±0.71^c^
PER *g/g* ^5^	3.18±0.79^a^	2.54±0.21^ab^	2.33 ±0.92^ab^	1.95±0.31^b^	1.69±0.41^b^

^abc^Different letter superscripts in the same row indicate significant difference (p0.05).

^1^Values = mean ± SD (n = 4).

^2^Diets: CNC = control with no added cholesterol; CC = Control with 2% cholesterol; LWS50 = 50% lyophilized washed sea cucumber + CC; CS50 = 50% cooked sea cucumber + CC; OS50 = 50% oven-cooked sea cucumber + CC.

^3^DM = Dry matter (grams per day).

^4^Initial weight = 140±11.35 g wet weight.

^5^Protein Efficiency Ratio = wet weight gain in grams / protein intake in grams.

Additionally, there is an error in the footnotes of Tables 4, 5, 7, and 8. “CC = Control with 1% cholesterol” should read “CC = Control with 2% cholesterol.” Please view the corrected Tables 4, 5, 7, and 8 here.

**Table 4:** Serum total triglycerides (Tg), total cholesterol (TC) and lipoproteins (LDL and HDL) levels, and the atherogenic index (AI) in rats fed a control or experimental diet containing sea cucumber (*I*. *badionotus*) meal.

	Diets^2^				
Parameters^1^	CNC	CC	LWS50	CS50	OS50
Tg *mg/dl*	38.19 ± 20.32^c^	83.98 ± 15.82^a^	78.37 ± 22.43^ab^	53.73 ± 18.70^b^	43.16 ± 10.57^c^
TC *mg/dl*	45.66 ± 6.58^d^	84.81 ± 10.0^a^	62.41 ± 5.0^bc^	55.19 ± 3.49^cd^	68.41 ± 6.52^b^
HDL *mg/dl*	50.94 ± 6.37^a^	50.54 ± 6.17^a^	43.81 ±3.67^ab^	41.38± 2.25^b^	44.62 ± 4.96^ab^
LDL *mg/dl*	8.89 ± 3.98^d^	49.55 ± 9.40^a^	23.17 ± 4.79^c^	20.89 ± 2.87^c^	33.11 ± 5.71^b^
AI^3^ *LDL/HDL*	0.17 ± 0.06 ^d^	0.98 ± 0.09 ^a^	0.52 ± 0.04 ^c^	0.50 ± 0.02 ^c^	0.74 ± 0.04 ^b^

^1^Values = means (n = 4) ± SD, in milligrams / deciliter;

^abcd^Different letter superscripts in the same row indicate statistical difference (p<0.05).

^2^CNC = control no added cholesterol; CC = Control with 2% cholesterol; LWS50 = 50% lyophilized washed sea cucumber + CC; CS50 = 50% cooked sea cucumber + CC; OS50 = 50% oven-cooked sea cucumber + CC;

^3^Atherogenic index.

**Table 5:** Liver total triglycerides (Tg), total cholesterol (TC) and total lipids (TL) levels in rats fed a control or experimental diet containing sea cucumber (*I*. *badionotus*) meal.

Parameters^1^	Diets^2^				
	CNC	CC	LWS50	CS50	OS50
Tg *mg/g dry liv*	46.81± 4.74^c^	89.97± 5.5^a^	60.22± 7.96^b^	47.59±6.17^c^	65.95±7.94^b^
TC *mg/g dry liv*	3.07 ± 0.23^b^	13.78 ± 4.29^a^	4.50 ± 1.25^b^	12.01 ± 1.75^a^	10.89 ± 1.7^a^
TL *mg/g dry liv*	177.36± 41.5^c^	328.61 ± 35^ab^	196.79± 17^c^	378.82±70^a^	272.31 ± 35^b^

^1^Values = means (n = 4) ± SD, in milligrams / gram dry liver;

^abc^Different letter superscripts in the same row indicate statistical difference (p<0.05).

^2^CNC = control no added cholesterol; CC = Control with 2% cholesterol; LWS50 = 50% lyophilized washed sea cucumber + CC; CS50 = 50% cooked sea cucumber + CC; OS50 = 50% oven-cooked sea cucumber + CC.

**Table 7:** Fatty acid methyl ether (FAME) concentration in livers from rats fed control and experimental diets.

Fatty acids^1^	Diets^2^				
	CNC	CC	LWS50	CS50	OS50
C14:0	0.19± 0.05^c^	1.11±0.12 ^a^	0.23 ± 0.004^bc^	ND	0.33 ± 0.003^b^
C15:0	ND	ND	0.29± 0.09^a^	ND	0.32± 0.039^a^
C16:0	20.42 ± 3.4^d^	36.13 ± 3.13 ^a^	17.614± 6.88^cd^	33.0± 5.67^ab^	27.71 ± 6.43^bc^
C16:1n-9	1.70 ± 0.20^b^	10.35 ± 1.22^a^	4.08 ± 0.82^b^	8.68 ± 1.01^a^	10.43 ± 4.2^a^
C17:0	ND	ND	ND	ND	0.49± 0.091
C18:0	14.48 ± 2.85^a^	11.36 ± 2.79 ^a^	8.96 ± 2.36^c^	15.02 ± 1.2^ab^	12.24 ± 3.39^c^
C18:1n-9	11.39± 3.04^c^	47.43 ±10.56 ^b^	8.96 ± 2.36 ^d^	24.99 ± 5.19^b^	15.20 ± 7.8^c^
C18:2n-6	18.02 ±6.19^b^	33.49± 13.8 ^a^	18.04 ± 8.70^b^	46.15 ± 6.49^a^	41.59 ± 5.57^a^
C18:3n-6	ND	ND	0.278± 0.07	0.278± 0.07	1.18± 0.71
C20:0	ND	2.21± 0.50 ^a^	ND	ND	1.05± 0.71^b^
C20:1	ND	ND	ND	ND	0.25± 0.03^b^
C20:2	ND	ND	0.82± 0.3^a^	ND	0.87± 0.23^a^
C20:3	ND	ND	1.30± 0.06^b^	ND	2.33± 0.31 ^a^
C20:4n-3	8.96± 1.25^a^	7.04 ±0.05^a^	4.60± 0.71 ^b^	ND	7.53 ± 1.86^a^
C24:0	ND	ND	ND	ND	0.33± 0.03
C24:1	ND	ND	ND	ND	0.48± 0.11
C22:6n-3	2.37± 0.35^ab^	1.53±0.089^bc^	0.90± 0.17^c^	ND	2.09± 0.55^bc^
**Σ Fatty acids** ^3^	77.29	150.65	94.78	128.118	83.73
Σ SFA	35.09	51.57	24.55	61.88	44.43
Σ MUFA	13.09	58.17	18.81	62.38	30.62
Σ PUFA	31.22	39.41	23.07	55.68	23.98
Σ PUFA/ Σ SFA	0.88	0.76	0.93	0.89	0.54
Σ PUFA/ Σ MUFA	2.38	0.68	1.23	0.89	0.78

^1^Concentracion in g FAME/100 g sample; values = mean (n = 4)± standard deviation

^abcd^Different letter superscripts in the same row indicate statistical difference (p<0.05).

^2^CNC = control no added cholesterol; CC = Control with 2% cholesterol; LWS50 = 50% lyophilized washed sea cucumber + CC; CS50 = 50% cooked sea cucumber + CC; OS50 = 50% oven-cooked sea cucumber + CC.

^3^SFA: saturated fatty acids; MUFA: monounsaturated fatty acids; PUFA: polyunsaturated fatty acids;∑Fatty acid = ∑SFA + ∑MUFA +∑PUFA; ∑SFA = C14:0 + C16:0 + C18:0 + C20:0 21:0 + C22:0 + C23:0 + C24:0; ∑MUFA = C16:1 + C18:1n9t +C20:1+ C24:1; ∑PUFA = C18:2n6t + C18:3n-6 + C20:3+ C20:4n6 + C22:6n3.

**Table 8:** Degree of steatosis in the livers of rats fed a control or experimental diet containing sea cucumber (*I*. *badionotus*) meal.

Diets^1^	Normal (%)	Degree of Steatosis^2^			
		< 5%	5–33%	33–6%	> 66%
CNC	100	-	-	-	-
CC		40	-	20	40
LWS50	100	-	-	-	-
CS50	20	-	-	-	80
OS50	60	20	-	-	20

Values are the average of five replicates.

^1^CNC = control with no added cholesterol; CC = Control with 2% cholesterol; LWS50 = 50% lyophilized washed sea cucumber + CC; CS50 = 50% cooked sea cucumber + CC; OS50 = 50% oven-cooked sea cucumber + CC.

^2^Degree of steatosis diagnosis score: < 5%: minimal steatosis; 5–33%: moderate steatosis; 33–66%: high steatosis. > 66%: severe steatosis [21].

## References

[1] Olivera-Castillo L, Davalos A, Grant G, Valadez-Gonzalez N, Montero J, Barrera-Perez HAM, et al (2013) Diets Containing Sea Cucumber (*Isostichopus badionotus*) Meals Are Hypocholesterolemic in Young Rats. PLoS ONE 8(11): e79446 doi:10.1371/journal.pone.0079446 24260223 10.1371/journal.pone.0079446 PMC3834158

