# Correction: Diets Containing Sea Cucumber (*Isostichopus badionotus*) Meals Are Hypocholesterolemic in Young Rats

**DOI:** 10.1371/journal.pone.0125451

**Published:** 2015-04-10

**Authors:** Leticia Olivera-Castillo, Alberto Davalos, George Grant, Nina Valadez-Gonzalez, Jorge Montero, Hirian Alonso Moshe Barrera-Perez, Yasser Chim-Chi, Miguel Angel Olvera-Novoa, Víctor Ceja-Moreno, Pablo Acereto-Escoffie, Jorge Rubio-Piña, Rossanna Rodriguez-Canul

Fig 1 is incorrect in both the original article and the correction published on March 6, 2015. The CNC data points and CC data points are erroneously switched. The authors have provided a corrected version here.

**Fig 1:**
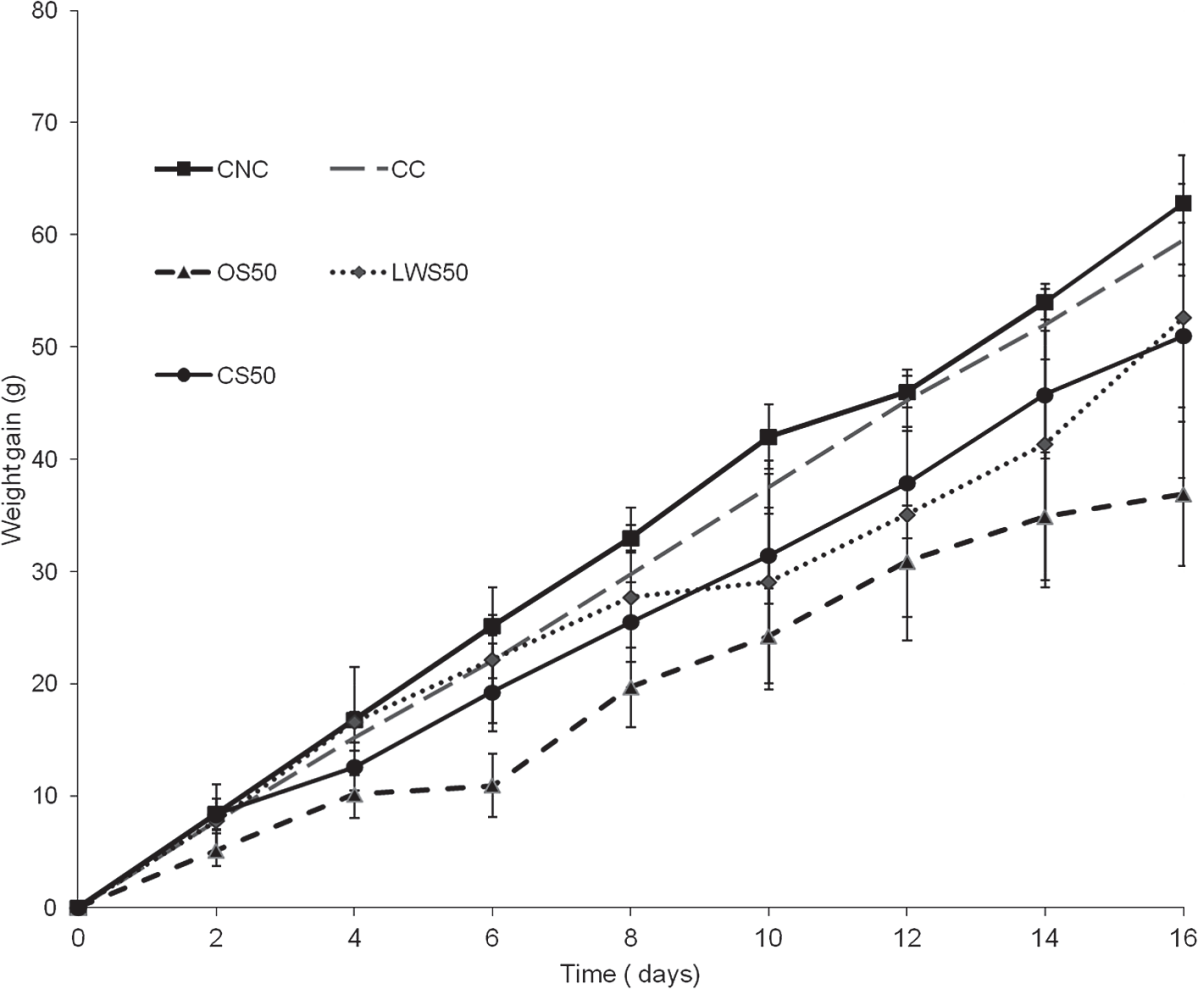
Weight gain in rats during dietary supplementation period. Growth during a 16-day period in rats (140 ± 11.35 g initial weight) fed equivalent daily amounts of a lactalbumin control diet with no added cholesterol (CNC); a lactalbumin control diet with 2% added cholesterol (CC); a diet containing 50% protein from cooked sea cucumber meal (CS50); one containing 50% protein from oven-cooked sea cucumber (OS50); or one containing 50% protein from lyophilized washed sea cucumber meal (LWS50). Values are means ± SD, N = 5.
