# Supplementary material for: Diets Containing Sea Cucumber (Isostichopus badionotus) Meals Are Hypocholesterolemic in Young Rats
Source: PLoS One. 2013 Nov 19;8(11):e79446. doi: 10.1371/journal.pone.0079446 (PMC3834158; doi:10.1371/journal.pone.0079446)
Supplement: Table S1 — (DOCX) [file pone.0079446.s001.docx]

**Table S1.** Primers used in this study for Real Time qPCR

| **Gene** | **Forward/ reverse primer (5´-3´)** |
| --- | --- |
| β- ACTIN | GCAGATGTGGATCAGCAAGC |
|  | GTCAAAGAAAGGGTGTAAAACG |
| HMGCR | GATGCAGCACAGAATGTGGG |
|  | TGCATTTTCTCCAGGATTGTC |
| LDLR | CTGGCGGCTGAGGAACATT |
|  | ATCCTCCAGGCTCACCATCT |
| FASN | GAGTATACAGCCACCGACCG |
|  | AGTTGCACACCACAAGGTCA |
| ABCA1 | AGCAGTTTGTGGCCCTCTTGT |
|  | TGAAGTTCCAGGTTGGGGTACTTG |
| ABCG1 | TCTCCGGGTTCTTTGTCAGC |
|  | ACCTCTCAGCCCGGATTTTG |
| APOA1 | CCTGGATGAATTCCAGGAGA |
|  | TCGCTGTAGAGCCCAAACTT |
| SCARB1 | CAAGAAGCCAAGCTGTAGGG |
|  | CCCAACAGGCTCTACTCAGC |
| CYP71a | CGCTATTCTCTGGGCATCTC |
|  | GTACCGGCAGGTCATTCAGT |
| APOB | TTCGAGAGCACCAAGTCCAC |
|  | GTATAGCACTCCGGCTGTCC |
| ME | CCCGCATCTCAACAAGGACT |
|  | CACGGTGGGAGTGTAAACGA |
| SERBP2 | ACAGCCAGTTACCATCCAGC |
|  | CAGCGTGGTCAAAACAAGGG |
| SERBP1C | CGCTACCGTTCCTCTATCAA |
|  | TTCGCAGGGTCAGGTTCTC |
| SERBP1a | TCCCAGAGTAGCCCCTTGTCC |
|  | CCAGTCCCCATCCACGAA |
| PPARGC1A | GCCACTACAGACACCGCACAC |
|  | ATTCGTCCCTCTTGAGCCTTTCG |
| PPARα | GAAGCAGATGACCTGGAAAGT |
|  | AGCCTGGACAGCTCCCTAA |
| PPARGC1A | GCCACTACAGACACCGCACAC |
|  | ATTCGTCCCTCTTGAGCCTTTCG |
| ACC | GTTGCACAAAAGGATTTCAG |
|  | CGCATTACCATGCTCCGCAC |
| CPT-1 | GCTTCCCCTTACTGGTTCC |
|  | AACTGGCAGGCAATGAGACT |
| G6PDH | GTTTGGCAGCGGCAACTAA |
|  | GGCATCACCCTGGTACAACTC |
| LXRA | AGGGCTGCAAGGGATTCTTC |
|  | GACACACTCCTCCCTCATGC |
| CROT | CGCCTACTTGGATGTGCGTA |
|  | TTCCAACTGAGTGCCTTCCC |
